# Yeast 26S proteasome nuclear import is coupled to nucleus-specific degradation of the karyopherin adaptor protein Sts1

**DOI:** 10.1038/s41598-024-52352-5

**Published:** 2024-01-24

**Authors:** Carolyn Allain Breckel, Zane M. Johnson, Christopher M. Hickey, Mark Hochstrasser

**Affiliations:** 1 Department of Molecular Biophysics and Biochemistry, Yale University, New Haven, CT 06520 USA; 2 Department of Molecular, Cellular, and Developmental Biology, Yale University, New Haven, CT 06520 USA; 3 Present Address: Arvinas, Inc., 5 Science Park, New Haven, CT USA

**Keywords:** Proteasome, Protein quality control, Protein translocation

## Abstract

In eukaryotes, the ubiquitin–proteasome system is an essential pathway for protein degradation and cellular homeostasis. 26S proteasomes concentrate in the nucleus of budding yeast *Saccharomyces cerevisiae* due to the essential import adaptor protein Sts1 and the karyopherin-α protein Srp1. Here, we show that Sts1 facilitates proteasome nuclear import by recruiting proteasomes to the karyopherin-α/β heterodimer. Following nuclear transport, the karyopherin proteins are likely separated from Sts1 through interaction with RanGTP in the nucleus. RanGTP-induced release of Sts1 from the karyopherin proteins initiates Sts1 proteasomal degradation in vitro. Sts1 undergoes karyopherin-mediated nuclear import in the absence of proteasome interaction, but Sts1 degradation in vivo is only observed when proteasomes successfully localize to the nucleus. Sts1 appears to function as a proteasome import factor during exponential growth only, as it is not found in proteasome storage granules (PSGs) during prolonged glucose starvation, nor does it appear to contribute to the rapid nuclear reimport of proteasomes following glucose refeeding and PSG dissipation. We propose that Sts1 acts as a single-turnover proteasome nuclear import factor by recruiting karyopherins for transport and undergoing subsequent RanGTP-initiated ubiquitin-independent proteasomal degradation in the nucleus.

## Introduction

The ubiquitin–proteasome system comprises a complex set of enzymes designed to degrade many different cellular proteins^1^. The proteasome is a ~ 2.5 MDa protein complex consisting of two major subcomplexes: the proteolytic 20S core particle (CP), and one or two 19S regulatory particles (RP). The RP is responsible for binding ubiquitin-tagged proteins and unfolding them for proteolysis in the CP; it is assembled from two smaller complexes known as the lid and base^2,3^. During exponential growth, most proteasomes are assembled into RP-CP complexes, known as 26S proteasomes^4^. Proteasome-mediated degradation generally occurs in an ATP- and ubiquitin-dependent manner, but a small number of proteasome substrates are known to undergo proteolysis without ubiquitin^5–7^.

Across eukaryotes, proteasomes often concentrate in the cell nucleus^4,8,9^. In *S. cerevisiae*, ~ 80% of proteasomes accumulate in the nucleus during exponential growth, and maintenance of this nuclear population is important to cell survival^4,10,11^. Several proteasome subunits in the RP base and CP contain classical nuclear localization signals (cNLSs), though it is not clear whether these NLS sequences are accessible in the 26S proteasome structure^12–15^. By contrast, the RP lid contains no known cNLS but still primarily localizes to the nucleus^16,17^. Although there is debate in the field about whether proteasomes are imported as various subcomplexes and later assembled in the nucleus, full 26S proteasomes are competent for karyopherin-mediated nuclear transport^4,11^.

Nuclear import is carried out by various nuclear transport receptors (NTRs) that ferry cargo proteins through the nuclear pore complex (NPC). For cNLS-containing proteins, the NTR karyopherin-$\alpha$ (Srp1 or Kap60 in yeast) binds to basic residues of the NLS and subsequently recruits the NTR karyopherin-$\beta$ (Kap95 in yeast)^18–21^. Karyopherin-$\beta$ mediates transport through the NPC central transporter^22–25^.

Inside the nucleus, the small GTPase Ran, which is primarily in its GTP-bound form, facilitates the release of karyopherin-$\beta$ from the $\alpha$/$\beta$-heterodimer complex^26,27^. Removal of karyopherin-$\beta$ triggers the release of the cargo protein from karyopherin-$\alpha$ inside the nucleus and is followed by recycling of the NTRs back to the cytoplasm for repeated rounds of nuclear import^28–30^. Both karyopherin-$\alpha$ and karyopherin-$\beta$ are essential for yeast viability^31^.

We previously showed that fully assembled 26S proteasomes in *S. cerevisiae* are transported to the nucleus using the adaptor protein Sts1^11^. Sts1 is an essential protein in yeast and has been implicated in several cellular processes^32–36^. While no structure of Sts1 has been solved, it bears strong sequence similarity to the proteasome nuclear anchor protein Cut8 from the fission yeast *Schizosaccharomyces pombe*^37,38^. Certain structural features of Sts1 can be inferred from the crystal structure of Cut8 and structure prediction methods^39–41^.

**Figure 1:**
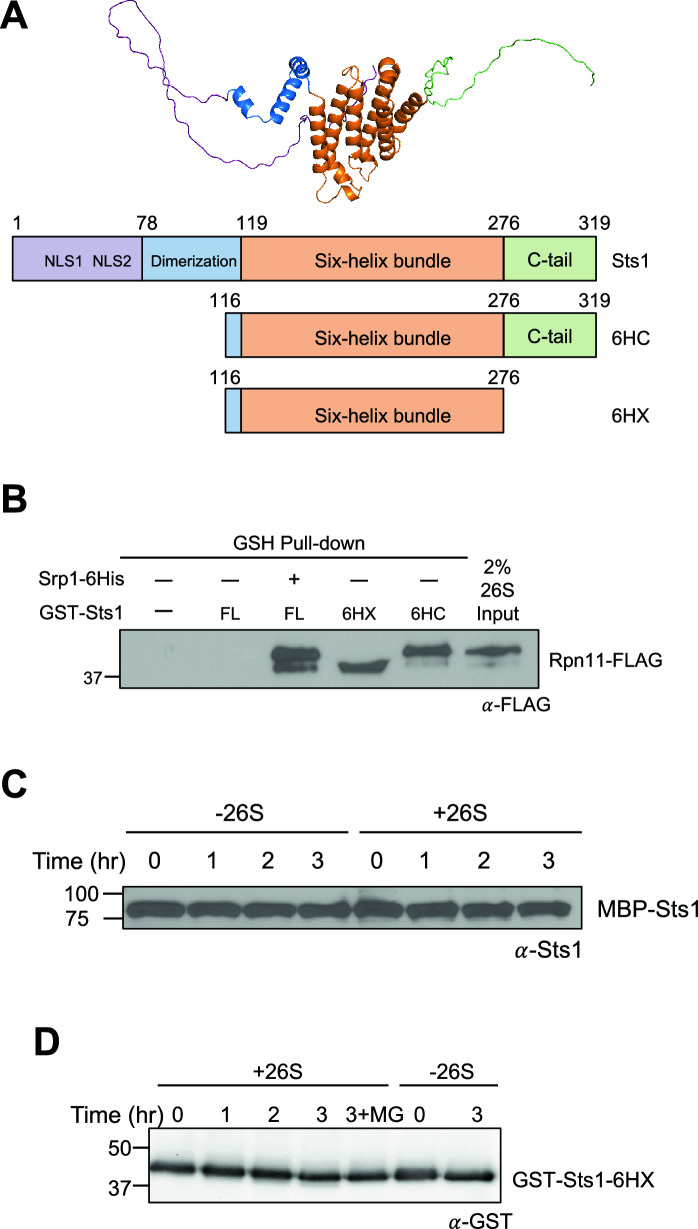
The Sts1 six-helix bundle is sufficient for proteasome interaction, but Sts1 degradation requires its N-terminus. (A) Predicted structure from AlphaFold2^39,41^ (top), and predicted domain architecture of Sts1 and the likely functional elements based on protein sequence analysis and comparison with the crystal structure of the *S. pombe* homolog Cut8 (bottom). “NLS1” and “NLS2” indicate the two basic segments of the bipartite nuclear localization signal. Both the N-terminus and C-terminus are likely to be unstructured. Truncation mutants Sts1(116–276) and Sts1(116–319) are represented as “6HX” and “6HC,” respectively. (B) The Sts1 six-helix bundle is sufficient for interaction with 26S proteasomes. Purified recombinant species of GST-Sts1, GST-Sts1/Srp1-6His, GST-Sts1(116–276), or GST-Sts1(116–319) were immobilized on a glutathione (GSH) resin and incubated with 26S proteasomes purified from yeast to detect interactions. “FL” indicates full-length GST-Sts1. 26S proteasome input represents 2% of incubated proteasomes. The migration of Rpn11-FLAG is distorted by the closely migrating 6HX GST fusion. (C) Sts1 degradation is not observed when the Sts1 N-terminus is blocked. In vitro degradation analysis of purified recombinant MBP-Sts1 by 26S proteasomes purified from yeast. Degradation was measured at room temperature. (D) The Sts1 six-helix bundle (when fused to GST) is not sufficient to initiate its proteasomal degradation in vitro. Degradation assay as in (**C**) conducted using purified recombinant GST-Sts1(116–276). For “3 + MG” sample, proteasomes were incubated with 50 μM MG132 proteasome inhibitor for 10 min prior to addition of Sts1. Images have been cropped for clarity and original blots are presented in Supplementary Fig. 5.

Sts1 contains a bipartite NLS sequence within its unstructured N-terminal domain that is sufficient for interaction with the yeast karyopherin-$\alpha$/Srp1^11^. Sts1 is also predicted to have two helical domains consisting of three and six helices, respectively (Fig. 1A), and likely assembles into a homodimer, as does Cut8^40^. Sts1 has been characterized as weakly binding to the proteasome lid subunit Rpn11 and is able to bind 26S proteasomes based on pulldown experiments^36,42,43^. Importantly, Sts1 is extremely short-lived and is a ubiquitin-independent substrate of the proteasome; degradation is blocked when it is complexed with Srp1^11^.

Here, we show that Sts1 facilitates nuclear import of the 26S proteasome in conjunction with the karyopherin-$\alpha$/$\beta$ heterodimer. Sts1 binds to karyopherin-$\alpha$ with its N-terminal NLS and to the 26S proteasome with its six-helix bundle region. The complex of Sts1 and the $\alpha$/$\beta$ heterodimer can be selectively disassembled by addition of RanGTP, but not the (cytoplasmic) GDP-bound form of Ran. Sts1 is a substrate of the proteasome, but its degradation appears to proceed only when its unstructured N-terminal domain is accessible; degradation is blocked when this domain is bound to the $\alpha$/$\beta$ heterodimer. Sts1 degradation in vitro is initiated when RanGTP removes the karyopherin proteins from Sts1. Importantly, cellular Sts1 is metabolically stabilized when proteasomes are excluded from the nucleus, indicating that its proteolysis is dependent upon successful completion of proteasome nuclear import in what appears to be a single-turnover import mechanism. We also show that Sts1-mediated proteasome transport is specific to exponentially growing cells, as Sts1 neither localizes to cytoplasmic proteasome storage granules (PSGs) during carbon starvation nor participates in the rapid nuclear reimport of proteasomes upon glucose refeeding.

## Results

### The Sts1 six-helix bundle is sufficient for 26S proteasome interaction

We previously found through in vitro pull-down assays that a complex of GST-Srp1 and Sts1-6His exhibits a binding preference for the full 26S proteasome over its subcomplexes^11^. To narrow down the proteasome binding domain within Sts1, we began with an AlphaFold2 prediction of Sts1 structure and comparisons to the crystal structure of its *S. pombe* homolog Cut8^39–41^. Sts1 is predicted to bear two $\alpha$-helical domains flanked by disordered domains at both its N- and C-termini (Fig. 1A). The arrangement of its predicted $\alpha$-helices closely resembles that of Cut8, and we infer that Sts1 may similarly homodimerize via the putative three-helix domain formed by residues 78–119.

As we have previously characterized an interaction between the Sts1 N-terminal NLS and Srp1, we searched for proteasome interaction sites within the C-terminal portion of the protein, specifically its six-helix-bundle domain and unstructured C-terminal tail. Recombinant GST-tagged Sts1 truncation mutants bearing only the six-helix bundle (residues 116–276, “6HX”) or the six-helix bundle with the C-terminal tail (residues 116–319, “6HC”) were immobilized on a glutathione resin and used in in vitro pull-down assays with 26S proteasomes purified from yeast. We compared these proteasome interactions to those of full-length GST-Sts1 (“FL”) or the complex of GST-Sts1/Srp1-6His. Both Sts1 truncation mutants bound purified proteasomes to a degree similar to the recombinant full-length GST-Sts1/Srp1-6His complex (Fig. 1B). These data demonstrate that the Sts1 six-helix bundle is sufficient for proteasome binding. This is consistent with previous results showing that a point mutation within the Sts1 six-helix bundle, C194Y, disrupted proteasome localization in vivo and proteasome interaction in vitro^11,35^. A recombinant truncation mutant lacking the six-helix bundle, Sts1(1–116)-6His, was unable to interact with 26S proteasomes when bound to GST-Srp1, consistent with the six-helix bundle being required for proteasome binding (Fig. S1A, B).

Unlike the N-terminally truncated Sts1 variants, recombinant full-length GST-Sts1 was unable to bind stably to proteasomes in vitro in the absence of Srp1. We note that in the AlphaFold2 model of Sts1, the N-terminus folds back onto the six-helix bundle, although this part of the model was of low confidence. Taken together, these results indicate that the Sts1 six-helix bundle domain is sufficient for proteasome recruitment but that Srp1 association with Sts1 may be required for this interaction to occur in full-length Sts1.

### The Sts1 N-terminus is required for its ubiquitin-independent proteolysis

Ubiquitin-independent degradation of proteins by the 26S proteasome requires RP subunits to bind to the substrate directly. Another feature of such substrates is a disordered domain that can enter the CP while the substrate is still bound to the RP^44,45^. Sts1 directly binds to the proteasome and possesses unstructured domains at both its N- and C-termini (Fig. 1A). We previously showed recombinant Sts1-6His is degraded by the proteasome in vitro within three hours, but proteolysis is blocked when Sts1 is pre-bound to Srp1, possibly by protecting its N-terminus from proteasomal engagement^11^. We hypothesized that the N-terminus initiates proteasomal degradation after Sts1 has bound to the RP, and that blocking this domain by fusing with a bulky protein would similarly block proteolysis in vitro. To test this, we fused MBP to the N-terminus of Sts1 and purified the fusion protein from *E. coli*. Coincubation of MBP-Sts1 with purified 26S proteasomes did not lead to a detectable decrease in MBP-Sts1 levels, suggesting that fusion of the disordered Sts1 N-terminus to MBP blocks Sts1 degradation (Fig. 1C).

It is possible that large tags such as MBP and GST may somehow block Sts1 interaction with the proteasome. In vivo, GST-Sts1-GFP was able to accumulate in the nucleus to a similar extent as Sts1-GFP, though GST-Sts1-GFP was present at much higher levels (Fig. S1C, S1D). However, GST-Sts1 in complex with Srp1 was still able to recruit the proteasome in vitro, and GST-Sts1-6HX, which has only the six-helix bundle of Sts1, also bound the proteasome (Fig. 1B). These results suggest proteasome binding is not sufficient for Sts1 degradation. This inference was supported by the finding that GST-Sts1-6HX was also not degraded by purified 26S proteasomes (Fig. 1D). These data indicate that Sts1 directly binds to the proteasome via its six-helix bundle domain and suggest that Sts1 ubiquitin-independent degradation requires access to its unstructured N-terminal domain.

### Sts1 forms a RanGTP-sensitive ternary complex with the karyopherin-$\alpha$/$\beta$ heterodimer

**Figure 2:**
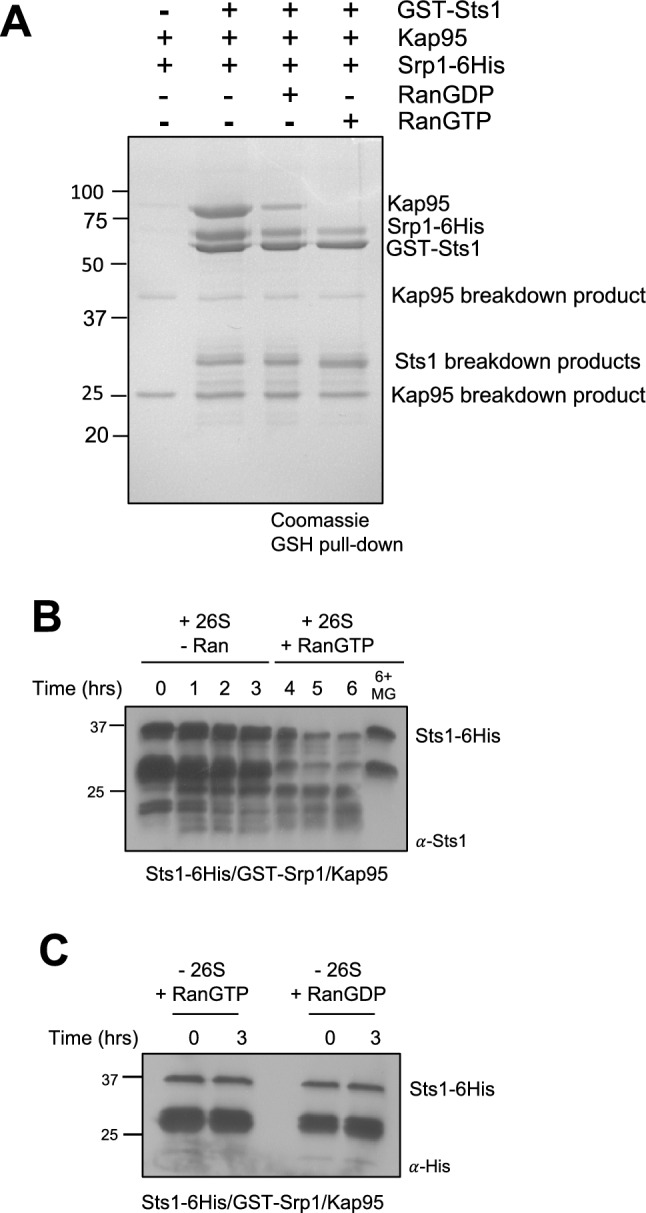
Sts1 proteasomal degradation correlates with removal of Kap95 by RanGTP. (**A**) Sts1 forms a ternary complex with Srp1 and Kap95 that can be selectively disrupted by the addition of RanGTP. Recombinant GST-Sts1 was immobilized on GSH resin and incubated with purified Kap95 and recombinant Srp1-6His to form a ternary complex. The complex was incubated with recombinant 6His-Gsp1GDP (RanGDP) or 6His-Gsp1GTP (RanGTP) to detect interactions and disassembly of the Sts1/Srp1/Kap95 complex. Pull-downs conducted at 4 °C. (**B**) Degradation of Sts1 is initiated when RanGTP removes karyopherin proteins from the Sts1 N-terminus. In vitro degradation assay as in Fig. 1C using the purified complex of recombinant Sts1-6His/GST-Srp1/Kap95 incubated with 26S proteasomes purified from yeast. After 3 h, purified recombinant RanGTP was added to the reaction mixture. For “6 + MG” sample, proteasomes were incubated with 50 μM MG132 proteasome inhibitor for 10 min prior to addition of the Sts1 complex. (**C**) Neither RanGTP nor RanGDP causes Sts1 instability in the absence of proteasomes. In vitro degradation assay as in (**B**), using the purified complex of Sts1-6His/GST-Srp1/Kap95 in the presence of purified RanGTP and RanGDP, respectively (as in **A**). Images have been cropped for clarity and original blots and gels are presented in Supplemental Fig. 5.

While a stoichiometric complex between Sts1 and Srp1/karyopherin-$\alpha$ had been demonstrated, we did not know if Sts1 could bind an Srp1/Kap95 (karyopherin-$\beta$) heterodimer, as predicted if Sts1 acts as an adaptor in proteasome nuclear import. To investigate this possibility, we utilized GST pull-down assays with recombinant GST-Sts1 immobilized on a glutathione resin. Both Srp1-6His and untagged Kap95 bound to GST-Sts1 (Fig. 2A). The relative amounts of Srp1-6His and Kap95 pulled down by GST-Sts1 were similar, consistent with the three proteins forming a ternary complex. This supports a model of Sts1-mediated nuclear import of proteasomes by a karyopherin-α/β-dependent mechanism.

We next examined whether the Sts1/Srp1/Kap95 ternary complex could be disrupted by GTP-bound Ran (yeast Gsp1). RanGTP in the nucleus displaces NLS-containing cargo from Srp1 and triggers the release of the karyopherin proteins for recycling back to the cytoplasm^27–30^. We would predict similar release of the karyopherin heterodimer from the Sts1 NLS in the presence of RanGTP. Following formation of the GST-Sts1/Srp1-6His/Kap95 ternary complex on a glutathione resin, we incubated the resin with either RanGTP or RanGDP, and examined what remained on the beads. The ternary complex remained largely intact upon exposure to RanGDP, whereas incubation with RanGTP triggered complete removal of Kap95 and a reduction in Srp1 (Fig. 2A). Maintenance of the full complex in the presence of RanGDP and persistence of the NLS-Srp1 interaction with RanGTP is consistent with what was previously observed with the Ulp1 SUMO protease and its classical bipartite NLS^46^. In those experiments, Kap95 was removed by RanGTP, but Srp1 was only removed from the Ulp1 NLS upon the concerted action of RanGTP and Exportin-2 (yeast Cse1)^46^. Further, it is possible that the presence of large affinity tags such as GST may impact the dissociation of Srp1 from the Sts1 N-terminus. We were unable to purify recombinant Cse1 from bacteria to test its contribution to the disassembly of the Sts1/Srp1/Kap95 complex. Together these data suggest that the Sts1/Srp1/Kap95 complex can stably form in the cytoplasm for recruitment of proteasome cargo and is likely disassembled in the nucleus through the action of RanGTP and potentially other factors after import.

### Sts1 ubiquitin-independent degradation is initiated by RanGTP

We hypothesized that removal of Kap95 and possibly Srp1 by RanGTP from Sts1 would normally occur in the nucleus after conveyance of 26S proteasomes by the import complex. Removal of the karyopherins would theoretically leave Sts1 bound to the proteasome with its disordered N-terminus available for threading into the proteasome core. We attempted to reconstitute this potential initiation event in vitro using purified components. We pre-formed the Sts1-6His/GST-Srp1/Kap95 ternary complex and analyzed the amount of Sts1 remaining in the presence of 26S proteasomes and RanGTP (Fig. 2B). As we had observed previously, in the absence of RanGTP, there was minimal degradation of Sts1-6His, likely due to the stability of the Sts1/Srp1/Kap95 complex. However, addition of RanGTP to the reaction mixture was sufficient to trigger Sts1-6His degradation. This degradation is proteasome-dependent, as no Sts1 proteolysis was observed in the presence of the proteasome inhibitor MG132, even after six hours of incubation with proteasomes. We note that RanGDP had variable effects in similar degradation assays, possibly due to contaminating RanGTP (Fig. S2). While Sts1-Srp1 binding appeared to remain stable with RanGTP (Fig. 2A), we suggest that association is altered in a way that allows degradation initiation, for example, by partial release of the bipartite NLS from Srp1.

To further ensure that the apparent degradation of Sts1 was not the result of decreased solubility in the presence of RanGTP, we also tested the stability of the Sts1/Srp1/Kap95 complex after several hours of incubation with either RanGTP or RanGDP but in the absence of the proteasome (Fig. 2C). With either form of Ran, no Sts1 depletion was observed, indicating that RanGTP does not affect Sts1 solubility or stability. These data suggest that the ternary complex formed between Sts1 and the karyopherins is specifically disrupted by RanGTP and precedes direct proteasomal degradation of Sts1.

### Proteasome-mediated degradation of Sts1 occurs preferentially in the nucleus

**Figure 3:**
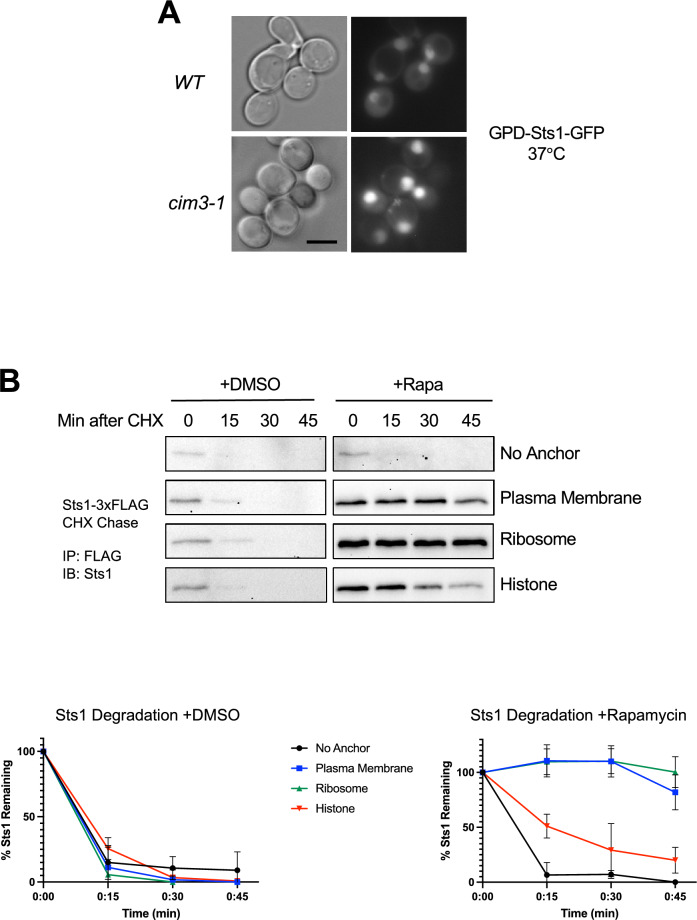
Sts1 proteasomal degradation occurs in the cell nucleus following nuclear import. (**A**) Sts1 accumulates in the cell nucleus when proteasomes are catalytically inactive. Wild-type and catalytically inactive proteasome mutant *cim3-1* yeast expressing the plasmid pRS415-GPD-Sts1-GFP were visualized by fluorescence microscopy at the restrictive temperature for *cim3-1*. Scale bar, 5 μm. (**B**) Sts1 degradation occurs when proteasomes are sequestered inside the nucleus but not when sequestered at the plasma membrane or ribosome. Using the Anchor Away yeast system, 26S proteasomes were anchored to the plasma membrane, ribosome, or chromatin. Each anchoring strain, as well as a control strain with no proteasome anchor, also bears chromosomally tagged *STS1-3xFLAG*. Cycloheximide-chase analysis was performed to determine the degradation rates of Sts1-3xFLAG in the presence (+ Rapa) or absence (+ DMSO) of proteasome sequestration in different cellular compartments. Cells were grown at 30 °C, treated with 10 μg/mL of either rapamycin or DMSO for two hours, and cycloheximide was added at time 0 to block further protein synthesis. FLAG immunoprecipitation was performed on cell extracts to enrich for Sts1-3xFLAG. Bottom panels: quantification of cycloheximide-chase data from at least three experiments in the presence (right) or absence (left) of proteasome sequestration. Images have been cropped for clarity and original blots are presented in Supplemental Fig. 5.

Initiation of degradation by RanGTP in vitro suggests Sts1 degradation may occur largely after the import complex reaches the nucleus. We first examined whether Sts1 accumulates in the nucleus when its degradation is blocked by proteasomal mutation^11^. We expressed a wild-type (WT) Sts1-GFP fusion protein in the temperature-sensitive yeast proteasome mutant *cim3-1* (*rpt6-1*)^48^. Sts1 continued to concentrate in the nucleus in *cim3-1* cells at restrictive temperature (Fig. 3A).

To determine whether Sts1 degradation relies on the presence of proteasomes in the nucleus, and thus the success of Sts1-mediated nuclear import, we utilized the Anchor Away technique to sequester the proteasome in different cellular compartments^10,49,50^. The plasma membrane protein Pma1, large ribosomal subunit Rpl13A, and histone H2B were each chromosomally fused to an FKBP12 protein domain. Additionally, the proteasome lid subunit Rpn11 was fused at its C-terminus to an FRB domain and GFP^10^. The FKBP12 and FRB domains can bind the compound rapamycin simultaneously; hence, in the presence of rapamycin, proteasomes become tethered to the plasma membrane, ribosomes, or chromatin (Fig. S3A,B). We conducted cycloheximide-chase analysis of chromosomally expressed Sts1-3xFLAG under each sequestration condition. Sts1 was immunoprecipitated via the FLAG affinity tag to concentrate sufficient protein for detection by anti-Sts1 immunoblotting. In the absence of rapamycin (DMSO control), Sts1-3xFLAG was rapidly degraded in all cases, exhibiting a half-life of less than 10 min, consistent with earlier results^11^.

Rapamycin treatment strongly stabilized Sts1 when proteasomes were anchored at the plasma membrane or ribosome, implying that Sts1 cannot be degraded efficiently in the cytoplasm (Fig. 3B, upper panel). By contrast, when proteasomes were anchored to chromatin via the histone H2B-FKBP12 fusion, substantial Sts1 degradation continued, albeit not quite as rapidly as in the “no anchor” control; tethering proteasomes to chromatin may slightly disrupt proteasome function or access to Sts1. Radioactive pulse-chase analysis of endogenous Sts1 under the same tethering conditions confirmed the trends seen by cycloheximide-chase analysis (Fig. S3C). Importantly, experiments with control proteasome substrates that build up in either the cytoplasm or nucleus demonstrated continued degradation when proteasomes were anchored in either compartment (Fig. S3D)^51^. The results indicated that all the different anchored proteasomes remained proteolytically functional (and suggested that these control substrates have sufficient access to all of them to support similar rates of degradation). We conclude that Sts1 degradation only proceeds efficiently when proteasomes are in the cell nucleus.

**Figure 4:**
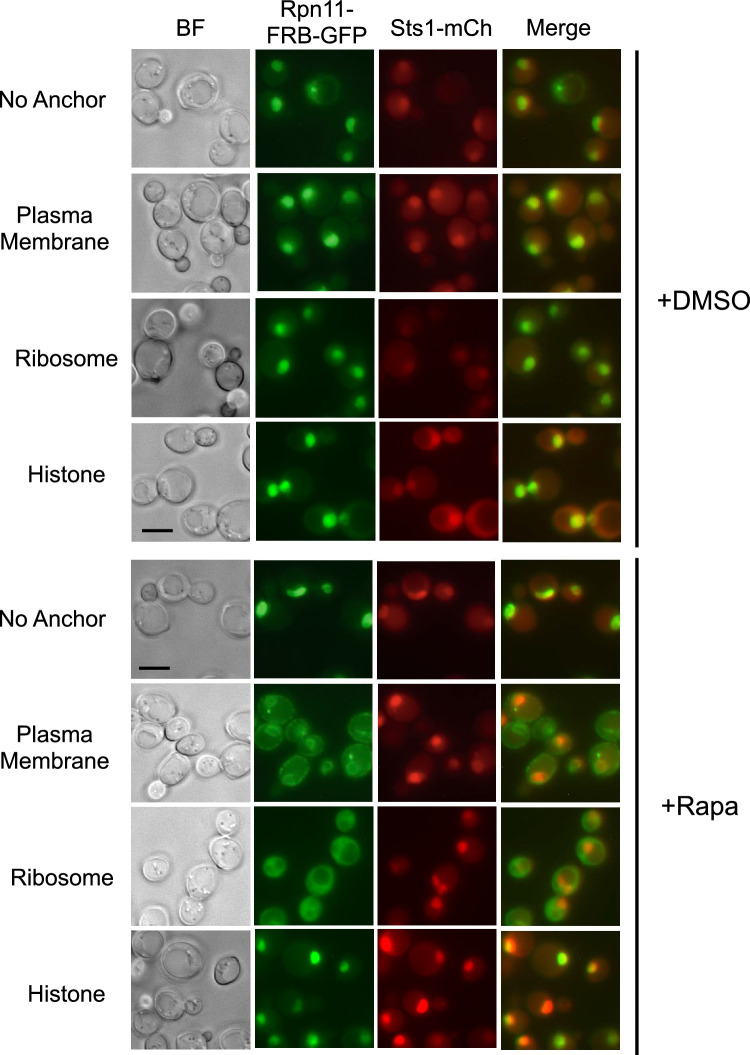
Sts1 can undergo nuclear import without binding proteasomes and accumulates in the nucleus even with proteasomes sequestered in the cytoplasm. The Anchor Away strains used in Fig. 3B were transformed with pRS415-MET25-Sts1-mCherry-FLAG plasmid to visualize Sts1 localization during proteasome sequestration (via chromosomally tagged *RPN11-FRB-GFP*) by fluorescence microscopy after 3 h of rapamycin or DMSO treatment. Cells were grown and treated with either DMSO or rapamycin as in Fig. 3B. Scale bar, 5 μm. Images have been false-colored and cropped for clarity.

Sts1 could be stabilized when proteasomes are tethered in the cytoplasm either because it continues to concentrate in the nucleus while proteasomes are depleted there, or because it is co-sequestered in the cytoplasm where strong binding of Srp1/Kap95 due to high RanGDP concentrations stabilizes it. To distinguish between these possibilities, we conducted fluorescence microscopy in each Anchor Away strain expressing chromosomally tagged *RPN11-FRB-GFP* and the *STS1-mCherry-FLAG* fusion on a plasmid under control of the *MET25* promoter. In each strain, both Rpn11-GFP and Sts1-mCherry localized to the cell nucleus with the vehicle control. However, when proteasomes were anchored to the plasma membrane or ribosome upon rapamycin treatment, Sts1-mCherry still predominantly localized to the nucleus despite proteasomal exclusion (Fig. 4). Thus, at least part of the observed Sts1 stabilization was likely due to a lack of proteasomes in the nucleus to degrade the Sts1 present there. These results also suggest that nuclear import of Sts1 can occur in the absence of proteasome cargo, consistent with the presence of an NLS in the Sts1 adaptor. This may also suggest that recruitment of karyopherin proteins in the cytoplasm occurs prior to proteasome binding, consistent with the inability of GST-Sts1 to pull down 26S proteasomes in the absence of Srp1 (Fig. 1B).

### Sts1 does not participate in reimport of proteasomes from PSGs

The role of Sts1 in karyopherin-mediated proteasome import led us to investigate if it has a similar role in the reimport of proteasomes from proteasome storage granules (PSGs). When budding yeast are in prolonged stationary phase or undergo glucose starvation, most proteasomes relocalize from the nucleus to the cytoplasm where they form PSGs^8,52^. PSGs persist until reintroduction of glucose causes their dissipation and rapid nuclear reimport of proteasomes^8,53^.

**Figure 5:**
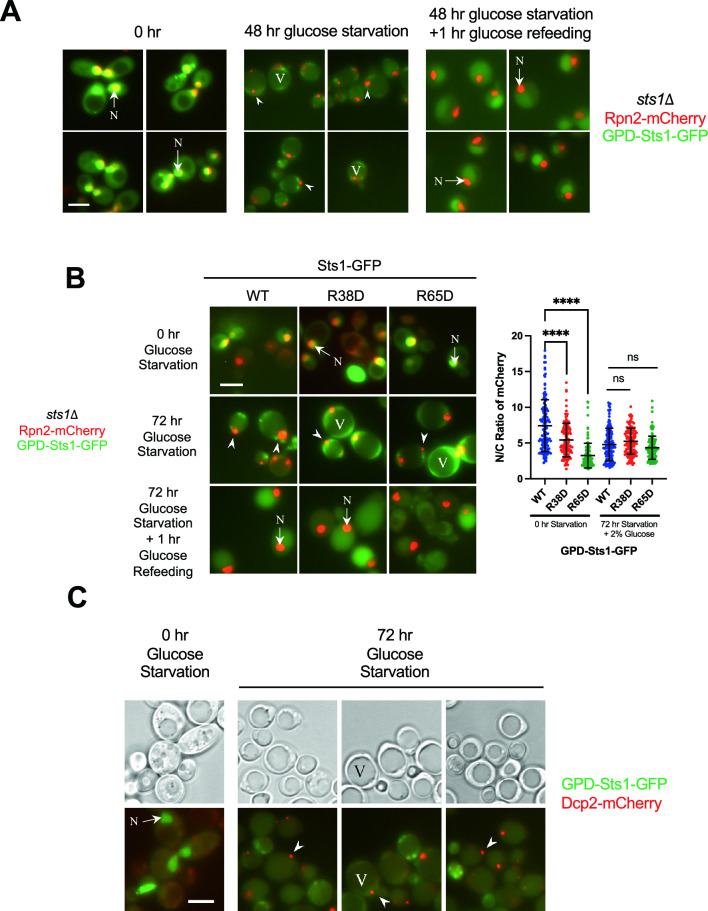
Sts1 not detected in proteasome storage granules (PSGs) or P-bodies under glucose starvation and does not participate in reimport of proteasomes from PSGs. (**A**) Sts1 does not localize to PSGs during glucose starvation and is not found in nuclei following reimport of proteasomes from PSGs. Yeast bearing the *sts1∆* mutation and chromosomal *RPN2-mCherry* were transformed with plasmid pRS415-GPD-Sts1-GFP for fluorescence microscopy. Cells were grown in rich medium (2% glucose) at 30 °C and imaged (0 h of glucose starvation). Cells harvested and subsequently grown in low-glucose medium (0.025% glucose) for 2 days and imaged, then supplemented with 2% glucose and imaged after 1 h of glucose refeeding. (**B**) Import-defective Sts1 mutants do not exhibit a proteasome localization defect following reimport of proteasomes from PSGs. Yeast described in panel A were transformed with plasmids pRS415-GPD-Sts1-GFP, pRS415-GPD-Sts1(R38D)-GFP, or pRS415-GPD-Sts1(R65D)-GFP for fluorescence microscopy. Cells were treated as in panel A. For quantification, at least 100 cells were counted from three replicates to determine the nucleus to cytoplasm ratio (N/C ratio) of Rpn2-mCherry in cells (right panel). A t-test was used to determine the statistical significance of differences in localization (****p < 0.0001, “ns” indicates no significant difference). (**C**) Sts1 does not localize to P-bodies upon glucose starvation. Yeast bearing chromosomally tagged *DCP2-mCherry* were transformed with plasmid pRS415-GPD-Sts1-GFP for fluorescence microscopy. Cells were grown in rich media and transferred to low-glucose media as in panel A for three days and imaged. Scale bars, 5 μm. Arrowheads indicate either PSGs (**A** and **B**) or P-bodies (**C**). “V” marks the cell vacuole. “N” marks the cell nucleus. Images have been false-colored and cropped for clarity.

To assess a potential role for Sts1 in nuclear reimport of proteasomes, we first localized Sts1-GFP after two days of glucose starvation and following one hour of glucose refeeding. During exponential growth, both Sts1-GFP and the proteasome marker Rpn2-mCherry concentrated in the nucleus (Fig. 5A). After glucose starvation, Rpn2-mCherry had relocalized to cytoplasmic PSG foci, but no Sts1-GFP signal was detected there. Following addition of glucose, Rpn2-mCherry again became highly concentrated in the nucleus, whereas Sts1-GFP was not detected there (Fig. 5A). Based on anti-Sts1 immunoblot analysis, Sts1-GFP was not detectably expressed during glucose starvation nor for at least one hour after glucose reintroduction (Fig. S4A). This was striking as proteasomes typically reenter the nucleus within minutes after glucose supplementation^8^. This suggested that Sts1 is not involved in the reimport of proteasomes from PSGs. In support of this, mutating the NLS elements in Sts1, which impairs proteasome import in exponentially growing cultures, had no impact on the reimport of proteasomes following glucose starvation and refeeding (Fig. 5B).

No Sts1-GFP signal was observed in PSGs during glucose starvation, but we often observed cytoplasmic puncta in the GFP fluorescence channel during glucose starvation (Fig. S4B). However, when we imaged yeast bearing no fluorescently tagged recombinant proteins, apparent cytoplasmic puncta were still visible in the GFP channel after three days of glucose starvation (Fig. S4C). Therefore, these puncta do not represent Sts1-GFP, consistent with our immunoblot analysis showing undetectable Sts1-GFP during glucose starvation and refeeding. Taken together, our data suggest that Sts1 neither localizes to PSGs nor contributes to nuclear reimport of proteasomes upon exit from quiescence.

## Discussion

**Figure 6:**
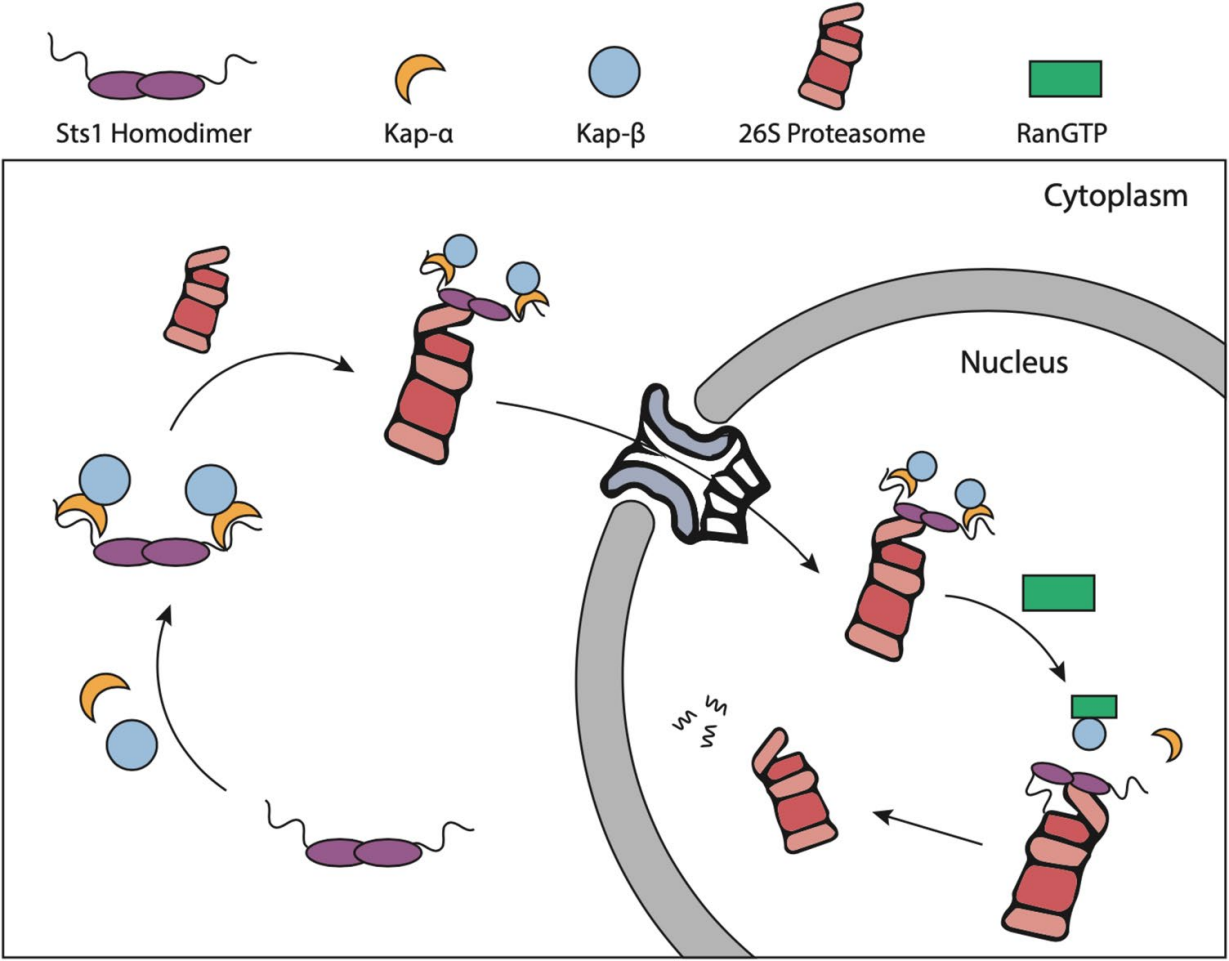
Model of Sts1/karyopherin-α/β-mediated nuclear import of 26S proteasomes and subsequent Sts1 ubiquitin-independent degradation. In the cytoplasm, Sts1, likely a homodimer, binds to karyopherin-α via interaction with the bipartite NLS sequence at the Sts1 N-terminus. Karyopherin-α recruits karyopherin-β and Sts1 subsequently binds to the 26S proteasome. This complex is imported into the nucleus via the nuclear pore complex. In the nucleus, RanGTP promotes removal of the karyopherin proteins from the Sts1 N-terminus. Once available, the unstructured Sts1 N-terminus is threaded into the proteasome RP and translocated into the CP to initiate ubiquitin-independent degradation of Sts1. This single turnover Sts1-mediated import mechanism occurs only during proliferative yeast growth.

Nuclear accumulation of proteasomes is broadly observed across eukaryotes, though the need for high relative proteasome levels in the nucleus is not well understood^4,9,54,55^. The contribution of the ubiquitin–proteasome system to DNA replication and repair, as well as the abundance of nuclear substrates such as transcription factors, likely contribute to the cellular demand for abundant nuclear proteasomes^56,57^. The Sts1 adaptor is responsible for mediating yeast proteasome nuclear import through interaction with Srp1^11,42^. Here, we provide evidence that proteasome nuclear import is facilitated by Sts1 through cooperation with the Srp1/Kap95 heterodimer and the RanGTP cycle. Most importantly, our results indicate that the ubiquitin-independent degradation of Sts1 by the proteasome requires successful nuclear import of proteasomes in vegetatively growing cells. This likely occurs through RanGTP-initiated nucleus-specific removal of the nuclear transport receptors from Sts1 that would otherwise shield it from degradation (Fig. 6).

The Sts1 six-helix bundle domain (residues 116–276) is sufficient for interaction with the proteasome (Fig. 1B). At the same time, the Sts1 N-terminal domain (residues 1–116) is sufficient for Srp1 binding, consistent with Sts1 bridging between the proteasome and karyopherin proteins^11^. Although the six-helix bundle is sufficient for proteasome binding, full-length Sts1 is incapable of recruiting the proteasome without Srp1. We have further demonstrated that Sts1 can form a ternary complex in vitro with Srp1 and Kap95 in the absence of proteasomes (Fig. 2A). This suggests that Sts1 NLS recognition by Srp1 and recruitment of Kap95 to Srp1 occurs prior to the Sts1-proteasome interaction. It is possible that the six-helix bundle domain is inaccessible or exists in an inhibited conformation prior to Srp1 recruitment, preventing an interaction with proteasomes until the nuclear transport complex has assembled. We demonstrated that Sts1 can accumulate in the nucleus even when the proteasome is sequestered in the cytoplasm (Fig. 4), suggesting that Sts1 is able to undergo import without associated proteasomal cargo.

Sts1 is a ubiquitin-independent substrate of the proteasome, and its degradation is blocked when Srp1 remains bound to the Sts1 N-terminus^11^. Our current data suggest Sts1 degradation relies upon the availability of its unstructured N-terminus to initiate degradation. Either blocking the N-terminus with fusion protein tags or removing the N-terminus prevents in vitro degradation of Sts1 by the 26S proteasome (Fig. 1C,D). These observations are consistent with previously characterized ubiquitin-independent proteasome substrates. Common features of such substrates include direct association with the proteasome and an unstructured region that can initiate degradation^44^. For Sts1, Srp1 binding to its disordered N-terminal domain may serve both to initiate nuclear transport and prevent premature Sts1 degradation prior to completion of proteasome nuclear import.

Sts1 can form a ternary complex with Srp1 and Kap95, and Kap95 association is selectively disrupted by the addition of RanGTP but not RanGDP (Fig. 2A). It is possible that the Sts1 bipartite NLS creates a particularly strong interaction that may not be fully disassembled by RanGTP alone; a potentially related example is the requirement for Exportin-2 for full dissociation of Srp1 from the Ulp1 bipartite NLS^46^. Although we were unable to examine the role of Exportin-2, our in vitro assays indicated that Sts1 degradation proceeds upon the addition of RanGTP alone (Fig. 2B). It is possible that partial dissociation of the bipartite NLS by RanGTP may already be sufficient to allow N-terminal capture and initiation of degradation by the 26S proteasome. In cells, Sts1 degradation would be licensed only after successful entry into nucleus and exposure to high RanGTP and possibly other factors such as Exportin-2 or Nup2^29,58^. The nuclear dependence of Sts1 degradation is strongly supported by our Anchor Away data, which demonstrate near complete inhibition of Sts1 degradation when proteasomes are sequestered in the cytoplasm (Fig. 3B).

Interestingly, Sts1 is likely not involved in the reimport of proteasomes from PSGs after exit from quiescence. Only a handful of proteasome-associated factors, specifically Blm10 and ubiquitin, have been identified as PSG components, and we did not observe Sts1 in PSGs (Fig. 5A)^59,60^. In fact, Sts1 is undetectable during glucose starvation and for at least an hour after glucose refeeding (Fig. S4A), yet proteasomes successfully reenter the nucleus minutes after glucose addition^8^. If proteasome nuclear import leads to the destruction of Sts1, it would necessitate continual *STS1* transcription and translation. While energetically costly, this mechanism would ensure the vectorial transport of fully assembled proteasomes into the nucleus without returning them to the cytoplasm if Sts1 also remained associated with Srp1 and was recycled with the transport receptor back to the cytoplasm. If Sts1 degradation is due to its proteasomal import partner, re-export might also serve as a quality control mechanism to ensure only active proteasomes are deposited in the nucleus. Such an energy-intensive import mechanism may not be favored during nutrient deprivation or early stages of growth recovery^61^.

Our results suggest a model of Sts1-mediated proteasome nuclear import and subsequent degradation that is inextricably linked to the karyopherin pathway (Fig. 6). In proliferating cells, karyopherin-$\alpha$ can recognize the Sts1 bipartite NLS sequence in the cytoplasm and recruit karyopherin-$\beta$. This may trigger a conformational change in Sts1 that allows binding to the 26S proteasome for karyopherin-mediated nuclear import. Inside the nucleus, binding of RanGTP to karyopherin-$\beta$ disrupts the import complex, freeing the Sts1 N-terminus for ubiquitin-independent proteasomal degradation.

An import mechanism with parallels to our model for Sts1 activity was recently suggested for the mammalian proteasome import adaptor AKIRIN2^62^. While structurally distinct and phylogenetically unrelated, AKIRIN2 similarly binds to 26S proteasomes and facilitates their nuclear import using an N-terminal bipartite NLS. AKIRIN2 also binds karyopherins and is a short-lived proteasome substrate. It remains to be seen if Cut8, the *S. pombe* ortholog of Sts1, functions similarly in nuclear import of proteasomes^37,38^.

## Materials and methods

### Yeast strain construction and growth

Table S1 includes the complete list of yeast strains used in this study. Yeast strains were created and maintained by a combination of mating followed by tetrad dissection, homologous recombination with PCR products, and plasmid transformation^63^. All strains are based on the MHY500 WT background unless otherwise noted with the exception of strains derived from the Anchor Away system which are in the W303 background^10^.

Yeast cells were grown in rich yeast-peptone-dextrose (YPD) or minimal (SD) media (2% glucose in either medium) at 30 °C unless otherwise noted. Other than the fluorescence microscopy experiments conducted under glucose starvation conditions (see below), all experiments were performed using exponentially growing yeast harvested at an OD_600_ between 0.8 and 1.2.

For glucose starvation, yeast cells were grown overnight at 30 °C in synthetic complete (SC) medium (0.67% yeast nitrogen base without amino acids, 0.5% casamino acids, 0.002% adenine, 0.004% tryptophan, 0.002% uracil, and 2% glucose)^65^. Drop-out medium lacking leucine was used for plasmid selection with pRS415-GPD-Sts1-GFP. Cells were diluted to 0.2 OD_600_ in fresh SC medium and grown to mid-log phase (OD_600_ between 0.8 and 1.2). Cells were centrifuged (5000 rpm, 5 min), rinsed with sterile water, and resuspended in SC medium containing 0.025% glucose (labeled as “Glucose Starvation” in figures) and cultured at 30 °C for 48–72 h, as indicated in the figure legends. For refeeding, cells that were grown in SC medium containing 0.025% glucose were supplemented with 2% glucose and grown for 1 h at 30 °C before harvesting.

### Plasmid constructions

A list of plasmids used in this study is provided in Table S2. All plasmids used were constructed using standard molecular genetic techniques. *STS1*- and *SRP1*-containing plasmids were created using a combination of restriction enzyme- or PCR-based cloning and site-directed mutagenesis. Constructs were verified by DNA sequencing. Yeast genomic DNA or other plasmids already in the Hochstrasser Lab database served as templates for cloning.

### Antibodies and immunoblotting

Immunoblotting was performed using the following primary antibodies: anti-GFP (JL8 antibody catalog no. 632380, Takara; 1:1000), anti-PGK (catalog no. 459250, Invitrogen; 1:20,000), anti-FLAG (F3165, Sigma; 1:10,000), anti-Sts1 (^11^; 1:5000), anti-Tetra-His (catalog no. 34670, Qiagen; 1:4000), anti-Rpn3 (catalog no. ab79769, Abcam; 1:5000), and anti-GST (catalog no. ab19256, Abcam; 1:10,000). Either donkey anti-rabbit IgG linked to horseradish peroxidase or sheep anti-mouse IgG linked to horseradish peroxidase (catalog no. NA934V and catalog no. NXA931V GE Healthcare, respectively) was used as the secondary antibody. Proteins were visualized on film (catalog no. E3018, Thermo Fisher Scientific) or with a G-box (SynGene) for quantification using enhanced chemiluminescence (ECL).

### Protein purification

Recombinant glutathione-S-transferase (GST), hexahistidine (6His), and maltose-binding protein (MBP) protein fusions were expressed and purified from Rosetta *E. coli* cells by their respective affinity tags using standard affinity purification methods^11^. Typically, 500 mL *E. coli* cultures were grown at 37 °C for 5 h until OD_600_ = 0.6 and induced with 0.4 μM IPTG at 16 °C for 18 h. Bacterial cell extracts were produced by sonication or using a French press in the presence of protease inhibitors (PMSF, cOmplete mini tablets [Roche]), followed by centrifugation (45,000×*g*, 35 min). Approximately 20 mL of cleared cell extract were applied to the appropriate resin for affinity tag-based purifications. Columns were washed extensively following binding of cell extracts in purification buffer, and purified proteins eluted using buffers containing l-glutathione, imidazole, or maltose, as appropriate. The purity of eluted species was assessed by SDS-PAGE and Coomassie Blue staining.

In the case of co-purified complexes comprising His-tagged and GST-tagged species, the individual plasmids were co-transformed into Rosetta *E. coli* cells (pET42b-based plasmids for His-tagged proteins and pGEX-6-P-1-based plasmids for GST-tagged proteins). The co-expressed complexes were grown and lysed as above and subsequently affinity purified using 6His-tag binding to TALON resin (Takara). Bound species were co-eluted with imidazole-containing buffer as above, and the purified complexes were assessed by SDS-PAGE and protein staining.

For the Sts1-6His/GST-Srp1/Kap95 complex, the Sts1-6His and GST-Srp1 proteins were co-expressed, and cells were lysed by sonication, followed by centrifugation (as above). Clarified cell extract was combined with lysed and clarified *E. coli* cell extract containing untagged Kap95. The full complex was then purified using 6His-tag binding to TALON resin. Purity of the complex was assessed by SDS-PAGE.

6His-Gsp1 bound to either GTP or GDP was purified according to the protocol outlined in Clarkson et al. (1996)^65^. Briefly, 6His-Gsp1 was expressed in Rosetta *E. coli* cells and was purified from cell extracts using Ni–NTA resin (Thermo Scientific) as above, and the eluted protein was aliquoted for nucleotide loading. 6His-Gsp1 was loaded with either 1 mM GDP or GTP in the presence of 5 mM EDTA to ensure exchange of nucleotide. Nucleotide-loaded Gsp1 was then dialyzed overnight at 4 °C and concentrated. Concentrated protein was purified by gel filtration using a Superdex S200 column.

26S proteasomes were affinity purified from yeast as described previously^66^. Briefly, yeast cells chromosomally tagged with *RPN11-3xFLAG* were grown in 2 L of rich medium (4% glucose) for 48 h. Harvested cells were frozen in liquid nitrogen and ground to a powder using a mortar and pestle in an ATP-containing lysis buffer. The cell powder was flash frozen in liquid nitrogen and smaller volumes of cell powder were thawed for each affinity purification. Typically, 20 mL of cell powder was thawed and resuspended in ATP-containing lysis buffer. Cell extracts were centrifuged, and the supernatant was applied to an anti-FLAG resin for 2 h. 26S proteasomes were then affinity-purified using 3xFLAG peptide to produce roughly 1 μM 26S proteasomes. The concentration and purification of proteasomes were evaluated by SDS-PAGE and Coomassie Blue staining using a BSA standard curve. To purify proteasome subcomplexes, yeast expressing Rpn11-3xFLAG was used to purify the 19S regulatory particle, and yeast expressing Pre1-3xFLAG was used to purify the 20S core particle^11^.

### Binding assays and pull-down assays with purified proteins

Analytical binding assays with 26S proteasomes affinity purified from yeast were conducted according to previously described protocols using various recombinant Sts1 species^11^. Pull-down assays using the karyopherin proteins were conducted according to a previously described protocol with slight modifications^46^. Briefly, recombinant GST-Sts1 was immobilized on 20 μL (packed volume) of glutathione (GSH) beads (equilibrated in binding buffer: PBS, 0.1% Tween-20, 0.2 mM DTT, fresh 0.2 mM PMSF). GST-Sts1 and resin were mixed and rotated at 4 °C for 1 h to bind the protein to the resin. Beads were centrifuged at room temperature (4000 rpm, 2 min), washed with 1 mL of binding buffer, and subsequently incubated with equimolar amounts of Srp1-6His and GST-cleaved Kap95 at 4 °C for 2 h on a rotator. Beads were centrifuged as before, washed once with 1 mL of binding buffer, and subsequently incubated with four-fold molar excess of 6His-Gsp1 (relative to Srp1-6His and Kap95) bound to the nucleotide GTP or GDP, as indicated (“RanGTP” and “RanGDP,” respectively in figures). Beads were incubated with these Gsp1 species at 4 °C for 2 h on a rotator. Beads were centrifuged as before and washed four times with 1 mL of binding buffer. Bound proteins were eluted from the GSH beads in 50 μL of 1X SDS gel sample buffer and analyzed by SDS-PAGE and protein staining.

### Degradation assays with purified proteins

Degradation analysis of purified proteins in vitro by proteasomes was conducted according to previously described protocols with slight modifications^11^. Recombinant prey species (MBP-Sts1, GST-Sts1(116–276), or Sts1-6His/GST-Srp1/Kap95) were incubated in the presence or absence of 26S proteasomes purified from yeast (assay buffer: 50 mM HEPES, pH 7.0, 150 mM NaCl, 10% glycerol, 6 mM MgCl_2_, 5 mM ATP, 0.1 mg/ml BSA). Reactions comprised final concentrations of 600 nM 26S proteasomes and 1.2 μM prey species. Reactions were incubated at room temperature with 20 μL fractions removed at the indicated intervals. Where indicated, reaction mixtures were supplemented with a two-fold molar excess of 6His-Gsp1 (compared to prey species) bound to either GTP or GDP. Fractions were centrifuged at room temperature (10,000 rpm, 2 min), the supernatant was separated from any precipitated material and mixed with gel sample buffer to stop the reaction. Fractions were placed on ice until the experiment was completed. Where indicated, a separate reaction of the prey species was tested in the absence of 26S proteasomes, or in the presence of 26S proteasomes that were pre-incubated for 10 min with 50 μM MG132 (Sigma Aldrich), a proteasome inhibitor, at room temperature. Supernatant fractions were resolved by SDS-PAGE and immunoblotted for Sts1 using anti-Sts1, anti-GST, or anti-His antibodies.

### Live cell microscopy and nuclear/cytoplasmic signal quantification

Cells were grown overnight in synthetic media (where indicated) at 30 °C, diluted to 0.2 and grown to mid-exponential phase (OD_600_ between 0.8 and 1.2). Culture aliquots of 1 mL were centrifuged, and cells were resuspended in 50 μL of the appropriate synthetic medium. Glass slides were spotted with 4 μL of cell suspension, a cover slip (18 mm × 18 mm, No. 1) was placed on top and sealed with nail polish, and samples were immediately imaged.

Imaging was performed on an Axioskop epifluorescence microscope (Carl Zeiss, Thornwood, NY) using a 100 × objective lens (plan-Apochromat 100 × /1.40 oil DIC) and an AxioCam MRm CCD camera (Carl Zeiss) with AxioVision software. All fluorescent images were captured using auto-exposure. After capture, the background was subtracted in ImageJ^67^, followed by quantification.

Quantification was performed using ImageJ. The summed signal intensities in equal-sized regions in the nucleus (N) and cytoplasm (C) of the same yeast cell were measured and the N/C ratio was determined using Microsoft Excel. Only cells with an identifiable nucleus (excluding cells that were clearly sick or dying or cells where the nucleus was not in the plane of focus) were counted. In all images where the vacuole was visualized, this region was avoided when taking measurements of cytoplasmic signal intensity.

Every experiment was repeated with three independent liquid-growth cultures of each strain or three independent plasmid transformants per strain. At least 100 yeast cells were quantified from each replicate. The difference in ratio of the nuclear to cytoplasmic signals between different strains or conditions was analyzed for statistical significance in GraphPad Prism8 by t-test.

### Cycloheximide-chase analysis

Protein degradation rates were determined by following a previously described protocol with slight modifications^68^. Cells were grown overnight at 30 °C in 5 mL of synthetic medium and diluted to 0.2 OD_600_ in 20 mL of medium. Where applicable, cells were treated with rapamycin or DMSO (vehicle control) according to the Anchor Away protocol described below. Once they reached mid-exponential phase (OD_600_ between 0.8 and 1.2), enough yeast cells were harvested to give 2.5 OD_600_ units per timepoint and resuspended in 7.5 mL medium and incubated for 5 min at 30 °C. Aliquots of 1 mL were harvested from each sample, and cycloheximide was added to each remaining culture volume to a final concentration of 0.25 mg/mL. Subsequent 1 mL aliquots were harvested from each culture at various intervals. Each 1 mL sample was added to 950 μL of ice-cold stop solution (30 mM sodium azide in water), followed by washing. Cells were lysed using an NaOH/SDS boiling method^47^. Briefly, cells equivalent to 2.0 OD_600_ units were harvested, centrifuged (10,000 rpm, 2 min), and washed with sterile water. Yeast cells were resuspended in 0.4 mL of 0.1 M NaOH, incubated at room temperature for 5 min, centrifuged as above, and resuspended in 100 μL of 1X gel loading buffer and frozen at -80 °C until use.

### Anchor Away yeast protein degradation assays

For cycloheximide-chase analysis of Sts1-3xFLAG in the different Anchor Away strains^10,51^, protein degradation rates were determined following previously described protocols with slight modifications^68^. Cells were grown overnight at 30 °C in 5 mL cultures in rich YPD medium and diluted to 0.2 OD_600_ in 40 mL of fresh medium. After approximately four hours of growth at 30 °C, cells were supplemented with either 10 μg/mL of rapamycin or DMSO (vehicle control) to allow specific subcellular sequestration of proteasomes as noted in the figures. Once cells reached mid-exponential phase (OD_600_ between 0.8 and 1.2) after roughly 6 h, 5 OD_600_ equivalents per timepoint were collected by centrifugation and resuspended in 5 mL culture medium (pre-warmed to 30 °C) and incubated for 5 min at 30 °C. 1 mL was harvested from each sample, followed immediately by addition of cycloheximide to the remainder of each culture at a final concentration of 0.25 mg/mL. Subsequent 1 mL aliquots were harvested at various intervals, centrifuged at room temperature (2 min, 10,000 rpm), and the resulting cell pellets were flash frozen in liquid nitrogen and stored at − 80 °C until use.

Cell pellets were thawed and resuspended in 325 μL of urea lysis buffer (50 mM Tris–HCl pH 7.5, 5 mM EDTA, 6 M urea, 0.5% SDS, 500 μM PMSF, 100 μM MG132). Roughly 100 μL of acid-washed glass beads (Sigma-Aldrich) were added to each sample, and cells were sheared using a bead beater for three cycles (1 min of bead beating at 4 °C, 2 min on ice). The sample tubes were then punctured using a 25G needle and centrifuged at room temperature to separate the cell extracts from the glass beads (4000 rpm, 2 min). To immunoprecipitate Sts1-3xFLAG from cell extracts, 20 μL (packed volume) of anti-FLAG M2 affinity gel (Sigma Aldrich) was added to 1.5 mL of wash buffer (150 mM NaCl, 30 mM HEPES pH 7.5, 5 mM EDTA, 0.2% Triton-X100, 0.1% SDS). 300 μL of each sample extract was added to the equilibrated anti-FLAG resin and rotated at 4 °C for 2 h. Samples were centrifuged at room temperature (4000 rpm, 2 min), and the pelleted beads were washed three times with 1 mL of wash buffer. Bound proteins were eluted from the anti-FLAG resin by heating for 5 min at 95 °C in 50 μL of 1X gel sample buffer and analyzed by immunoblotting with anti-Sts1. For quantification of cycloheximide-chase data, images digitally collected using a G-box system were processed using ImageJ. Quantifications represent the mean and standard deviation of three independent replicates.

For radioactive pulse-chase analysis, protein degradation rates were determined following previously described protocols with the addition of incubation in the presence of 10 μg/mL rapamycin or DMSO (as described above)^69^.

### Protein expression analysis during glucose starvation

Cells were grown overnight in 5 mL of synthetic complete medium (SC) as noted above, diluted to 0.2 OD_600_ in 20 mL fresh medium, and grown to mid-exponential phase (OD_600_ between 0.8 and 1.2). Cells were washed with sterile water and grown under glucose starvation conditions in SC medium containing 0.025% glucose for 72 h. Recovery from starvation was done by supplementing cells with 2% glucose and growing for an additional 7 h, as described above. Cells were lysed using the NaOH/SDS boiling method described above^47^. Yeast proteins from 0.1 OD_600_ units were resolved by SDS-PAGE followed by immunoblotting with antibodies against Sts1 and the loading control Pgk1 (phosphoglycerate kinase or PGK).

### Supplementary Information

Supplementary Figure S1.

Supplementary Figure S2.

Supplementary Figure S3.

Supplementary Figure S4.

Supplementary Figure S5.

Supplementary Tables.

## Data Availability

All data generated or analyzed during this study are included in this published article (and its Supplementary Information files).

## Abbreviations

CP: Core particle
RP: Regulatory particle
NLS: Nuclear localization signal
NTR: Nuclear transport receptor
NPC: Nuclear pore complex
WT: Wild type
N/C: Ratio of nuclear to cytoplasmic signal
GSH: Glutathione
YPD: Yeast-peptone-dextrose
SD: Synthetic defined
ECL: Enhanced chemiluminescence
OD_600_: Optical density (measured at wavelength 600 nm)
SDS: Sodium dodecyl sulfate
GST: Glutathione S-transferase
BSA: Bovine serum albumin
MBP: Maltose-binding protein
PSG: Proteasome storage granule
