# Supplementary material for: Yeast 26S proteasome nuclear import is coupled to nucleus-specific degradation of the karyopherin adaptor protein Sts1
Source: Sci Rep. 2024 Jan 24;14:2048. doi: 10.1038/s41598-024-52352-5 (PMC10808114; doi:10.1038/s41598-024-52352-5)
Supplement: Supplementary file 1 — Supplementary Figure S1. [file 41598_2024_52352_MOESM1_ESM.pdf]

Supplemental Figure 1

Breckel et al.

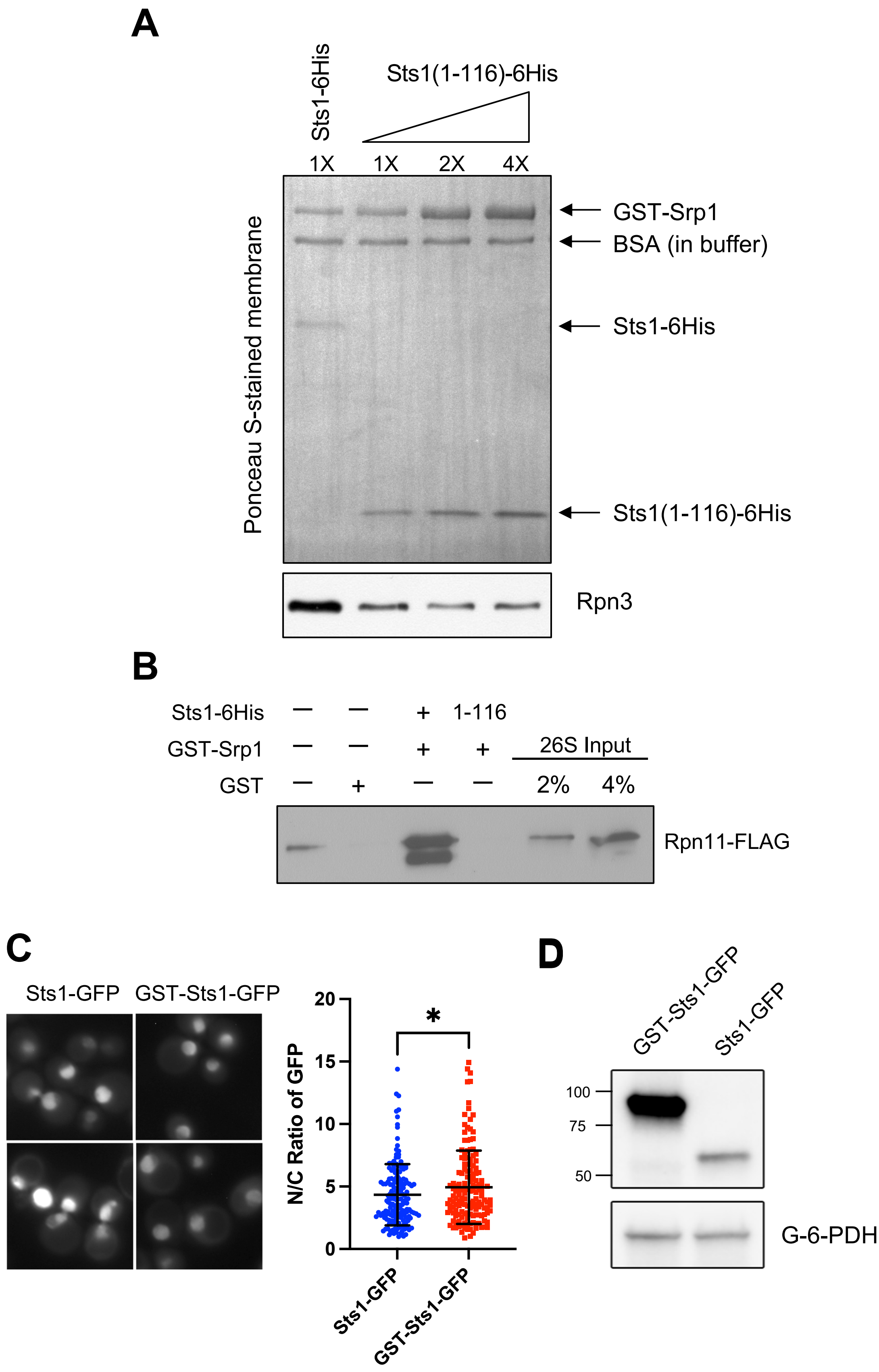

Figure S1. The Sts1 N-terminus is insufficient for interaction with the 26S proteasome. (A) Purified recombinant species of Sts1-6His/GST-Srp1 or Sts1(1-116)-6His/GST-Srp1 were immobilized on a glutathione (GSH) resin and incubated with 26S proteasomes purified from yeast to detect interactions. "1X," "2X," and "4X" indicate the relative amounts of the Sts1(1-116)-6His/GST-Srp1 complex that were loaded onto the glutathione beads in comparison to the Sts1-6His/GST-Srp1 complex. The upper panel represents a Ponceau S-stained membrane indicating the species in the bound complexes. The lower panel is an immunoblot against the proteasome subunit Rpn3. (B) Purified recombinant species of Sts1-6His/GST-Srp1, Sts1(1-116)-6His/GST-Srp1, or GST were immobilized on a glutathione (GSH) resin and incubated with 26S proteasomes purified from yeast to detect interactions. 26S proteasome input represents 2% or 4% of incubated proteasomes. (C) N-terminal affinity tags do not affect Sts1 localization. Yeast bearing the *sts1Δ* mutation were transformed with either pRS415-MET25-Sts1-GFP or pRS415-MET25-GST-Sts1-GFP for fluorescence microscopy. The cells were grown in rich medium at 30°C and imaged. For quantification, at least 100 cells were counted to determine the nucleus to cytoplasm ratio (N/C ratio) of Sts1-GFP or GST-Sts1—GFP in cells (right panel). A t-test was used to determine the statistical significance of differences in localization (*$p<0.1$). (D) GST-Sts1-GFP is stabilized compared to Sts1-GFP. Cells as described in (C) were harvested for Western extraction to determine their endogenous levels. G-6-PDH serves as a loading control. Images have been cropped for clarity and original blots are presented in Supplemental Figure 5.
