# Supplementary material for: Yeast 26S proteasome nuclear import is coupled to nucleus-specific degradation of the karyopherin adaptor protein Sts1
Source: Sci Rep. 2024 Jan 24;14:2048. doi: 10.1038/s41598-024-52352-5 (PMC10808114; doi:10.1038/s41598-024-52352-5)
Supplement: Supplementary file 2 — Supplementary Figure S2. [file 41598_2024_52352_MOESM2_ESM.pdf]

Breckel et al.

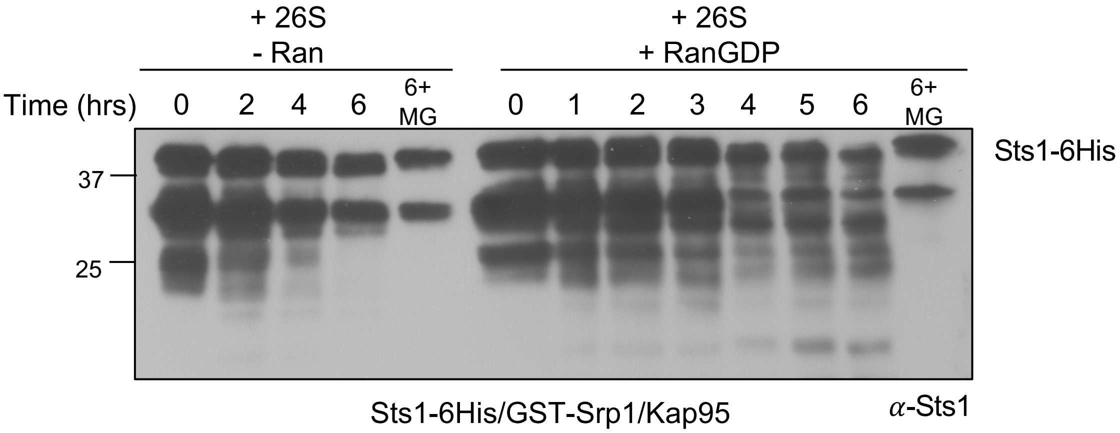

Figure S2. Sts1 ubiquitin-independent degradation initiation in the presence of RanGDP.

Degradation of Sts1 was assayed in the presence of RanGDP. In vitro degradation assay performed as in Fig. 1C using the purified complex of recombinant Sts1-6His/GST-Srp1/Kap95 incubated with 26S proteasomes purified from yeast. After 3 hrs, purified recombinant RanGDP was added to the reaction mixture. For "6+MG" samples, proteasomes were incubated with 50 μM MG132 proteasome inhibitor for 10 min prior to addition of the Sts1 complex. Original blot is presented in Supplemental Figure 5.
