# Supplementary material for: Yeast 26S proteasome nuclear import is coupled to nucleus-specific degradation of the karyopherin adaptor protein Sts1
Source: Sci Rep. 2024 Jan 24;14:2048. doi: 10.1038/s41598-024-52352-5 (PMC10808114; doi:10.1038/s41598-024-52352-5)
Supplement: Supplementary file 3 — Supplementary Figure S3. [file 41598_2024_52352_MOESM3_ESM.pdf]

Supplemental Figure 3

Breckel et al.

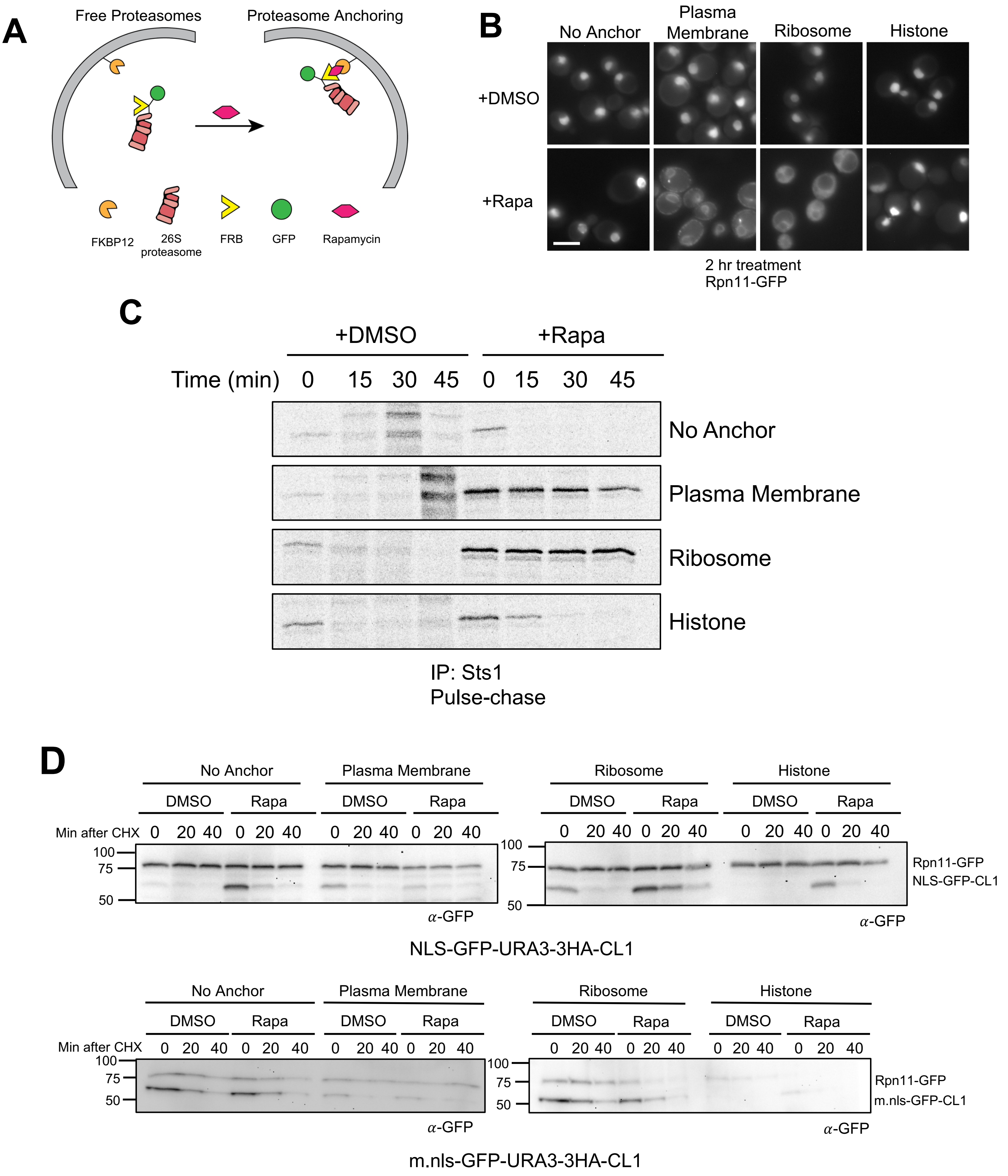

Figure S3. The Anchor Away system sequesters proteasomes to various cellular compartments and leads to differential degradation behavior of endogenous Sts1.
(A) Schematic of the Anchor Away system. The essential proteasome subunit Rpn11 is chromosomally tagged with FRB and GFP (*RPN11-FRB-GFP)* and properly localizes to the cell nucleus in the absence of rapamycin. An anchor protein is tagged with the FKBP12 protein such that in the presence of rapamycin a ternary complex will form between Anchor-FKBP12, rapamycin, and Rpn11-FRB-GFP. This will tether the fully assembled proteasome to the site of the anchor protein. (B) Representative examples of proteasomes sequestered to different cellular compartments in the Anchor Away system. Cells grown and treated with either DMSO or rapamycin as in Fig. 3B. Scale bar, 5 μm. (C) Sts1 degradation in the cell nucleus but not cytoplasm is also observed by radioactive pulse-chase analysis. Cells grown and treated with either DMSO or rapamycin as in Fig. 3B. [S35]Met/Cys pulse-chase analysis using Sts1 immunoprecipitation. (D) Sequestered proteasomes are active in the cytoplasm. Cycloheximide-chase analysis was performed with the Anchor Away yeast strains described in Fig. S3A expressing either the mostly nuclear degradation substrate NLS-GFP-URA3-3HA-CL1 or the mostly cytoplasmic substrate m.nls-GFP-URA3-3HA-CL1 from a plasmid. Cycloheximide-chase analysis was performed after a 2 hr incubation with rapamycin or DMSO. Images have been cropped for clarity and original blots and phoshorimages are presented in Supplemental Figure 5.
