# Supplementary material for: Yeast 26S proteasome nuclear import is coupled to nucleus-specific degradation of the karyopherin adaptor protein Sts1
Source: Sci Rep. 2024 Jan 24;14:2048. doi: 10.1038/s41598-024-52352-5 (PMC10808114; doi:10.1038/s41598-024-52352-5)
Supplement: Supplementary file 4 — Supplementary Figure S4. [file 41598_2024_52352_MOESM4_ESM.pdf]

Supplemental Figure 4

Breckel et al.

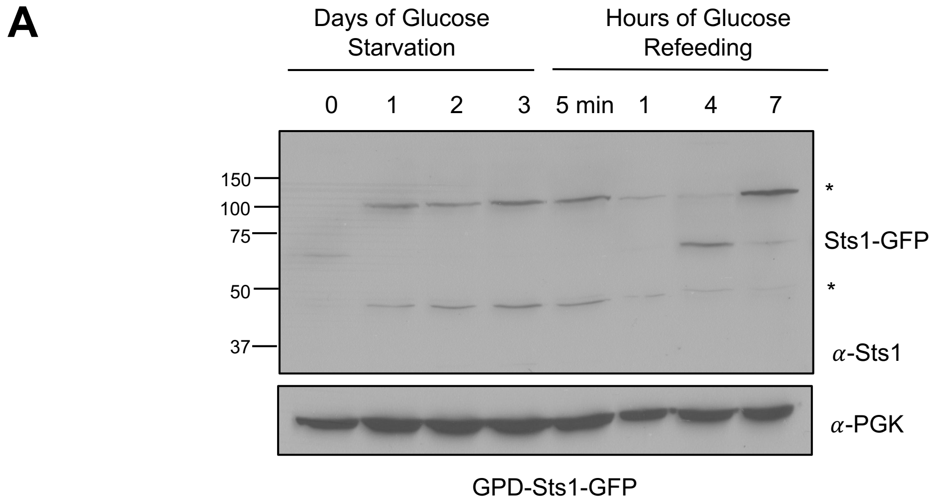

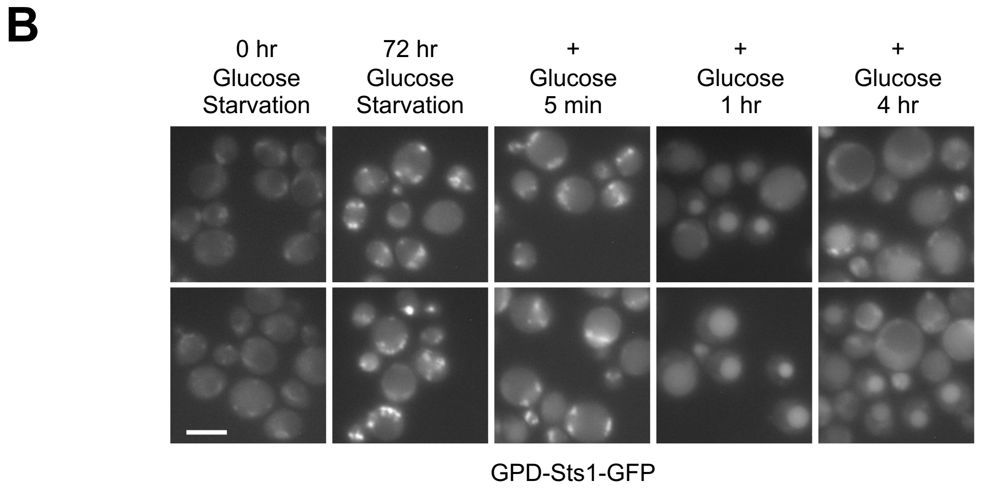

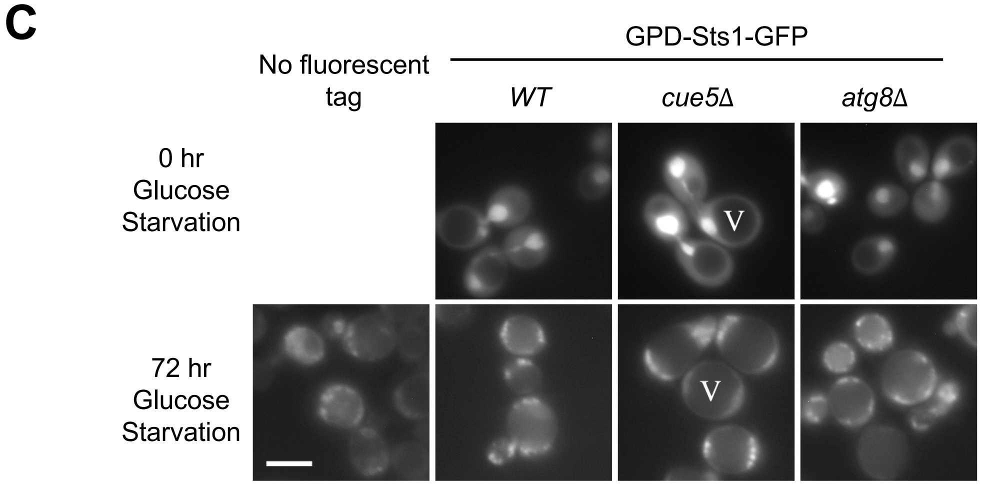

Figure S4. Glucose starvation conditions yield fluorescent cytoplasmic puncta that are not Sts1-GFP.

(A) Sts1 is not expressed during prolonged glucose starvation and is also not detected for at least 1 hr after glucose refeeding. Yeast bearing the plasmid pRS415-GPD-Sts1-GFP were grown in rich medium and transferred to low-glucose medium as in Fig. 5A for three days. Samples taken every 24 hrs and at the indicated timepoints after glucose refeeding for anti-Sts1 immunoblotting. "*" indicates non-specific cross-reactive species. (B) Cytoplasmic puncta are observed during glucose starvation that appear to localize to the vacuole upon glucose refeeding. Yeast bearing the plasmid pRS415-GPD-Sts1-GFP were treated as in panel A and imaged by fluorescence microscopy. (C) The cytoplasmic puncta observed under glucose starvation are not Sts1-GFP and are not in vacuoles. Yeast without a fluorescent label and yeast transformed with plasmid pRS415-GPD-Sts1-GFP were grown in rich media and glucose starved as in Fig. 5A for three days and imaged by fluorescence microscopy. "V" indicates cell vacuole. Scale bar, 5 μm. Images have been cropped for clarity and original blots are presented in Supplemental Figure 5.
