# Supplementary material for: Yeast 26S proteasome nuclear import is coupled to nucleus-specific degradation of the karyopherin adaptor protein Sts1
Source: Sci Rep. 2024 Jan 24;14:2048. doi: 10.1038/s41598-024-52352-5 (PMC10808114; doi:10.1038/s41598-024-52352-5)
Supplement: Supplementary file 5 — Supplementary Figure S5. [file 41598_2024_52352_MOESM5_ESM.pdf]

Supplemental Figure 5

Breckel et al.

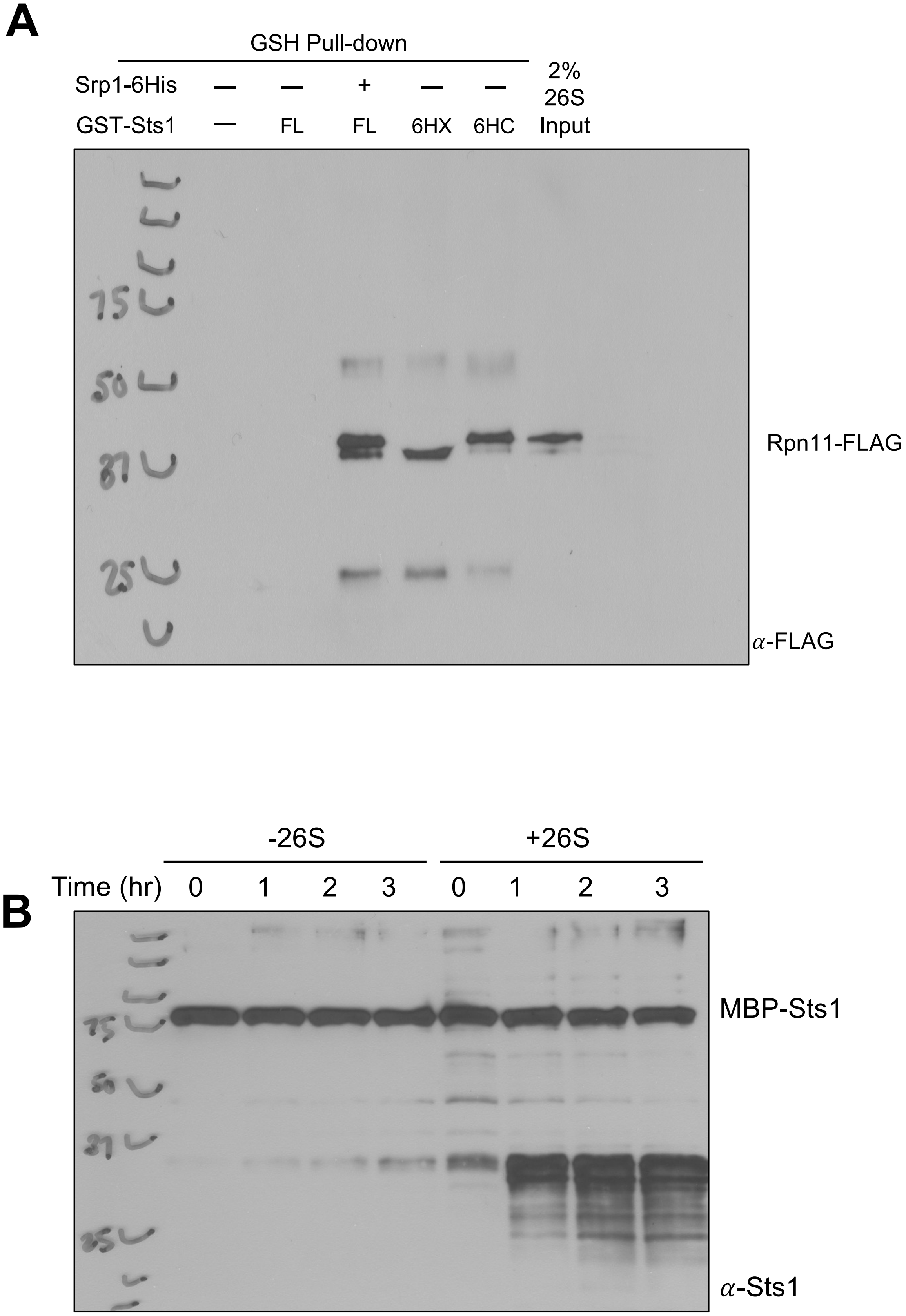

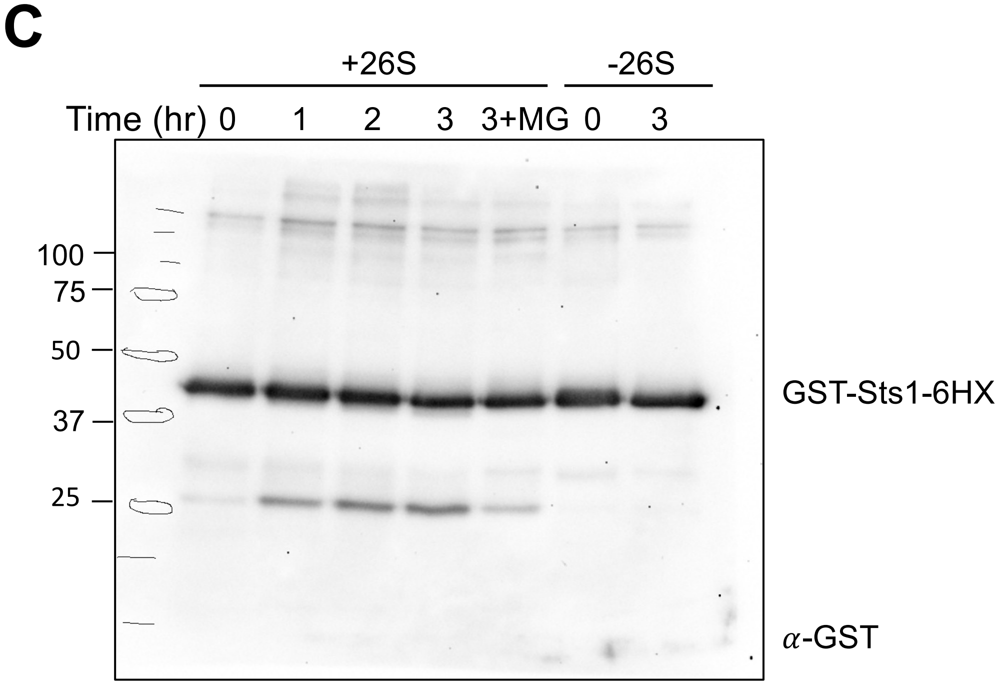
C
+26S
-26S
Time (hr) 0 1 2 3 3+MG 0 3
100
75
50
37
25
GST-Sts1-6HX
α-GST

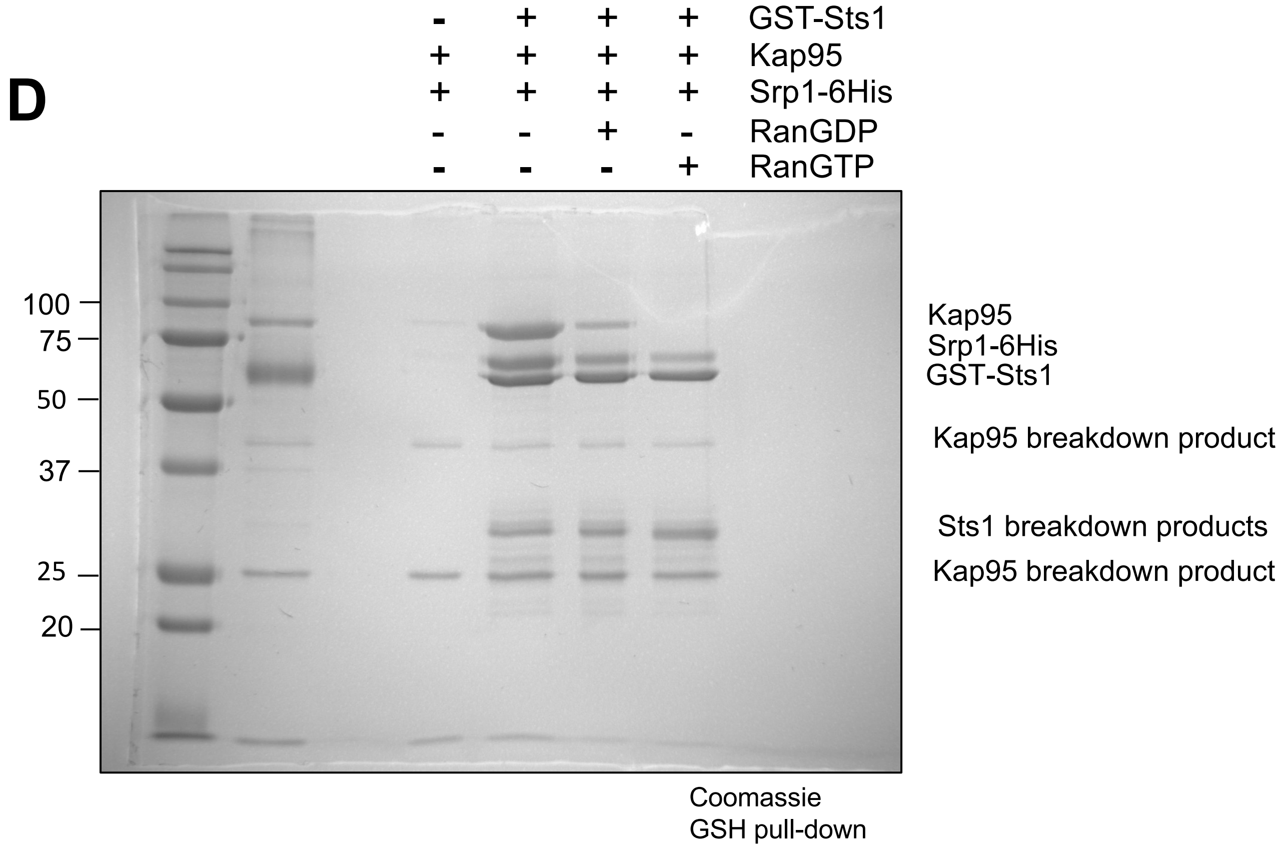
D
- + + + GST-Sts1
+ + + + Kap95
+ + + + Srp1-6His
- - + - RanGDP
- - - + RanGTP
100
75
50
37
25
20
Kap95
Srp1-6His
GST-Sts1
Kap95 breakdown product
Sts1 breakdown products
Kap95 breakdown product
Coomassie
GSH pull-down

E

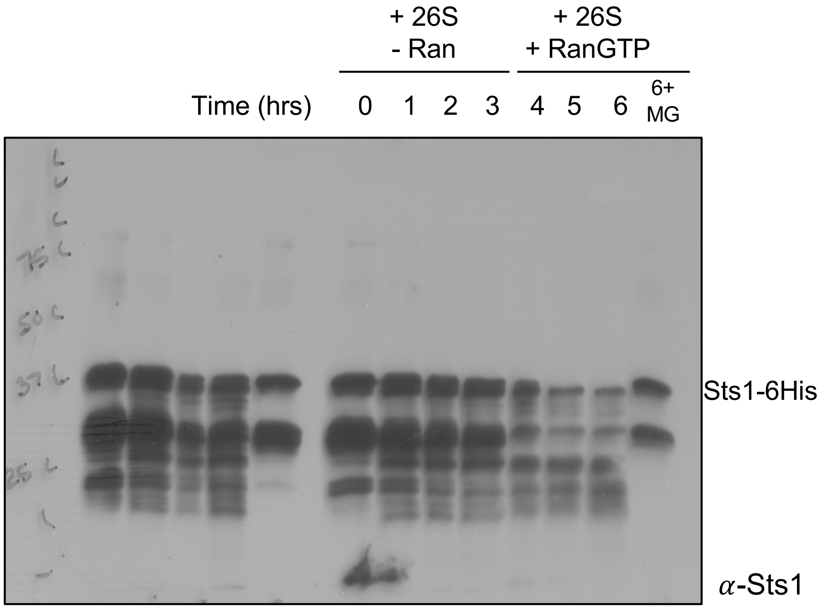

Sts1-6His/GST-Srp1/Kap95

F

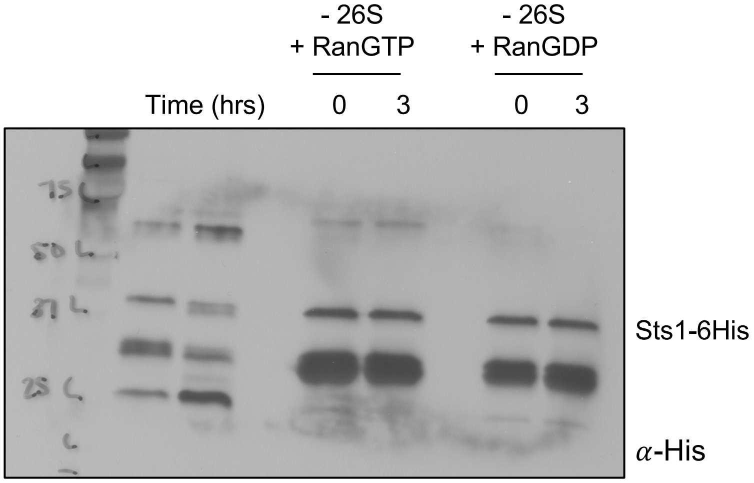

Sts1-6His/GST-Srp1/Kap95

Supplemental Figure 5 (cont.)

G

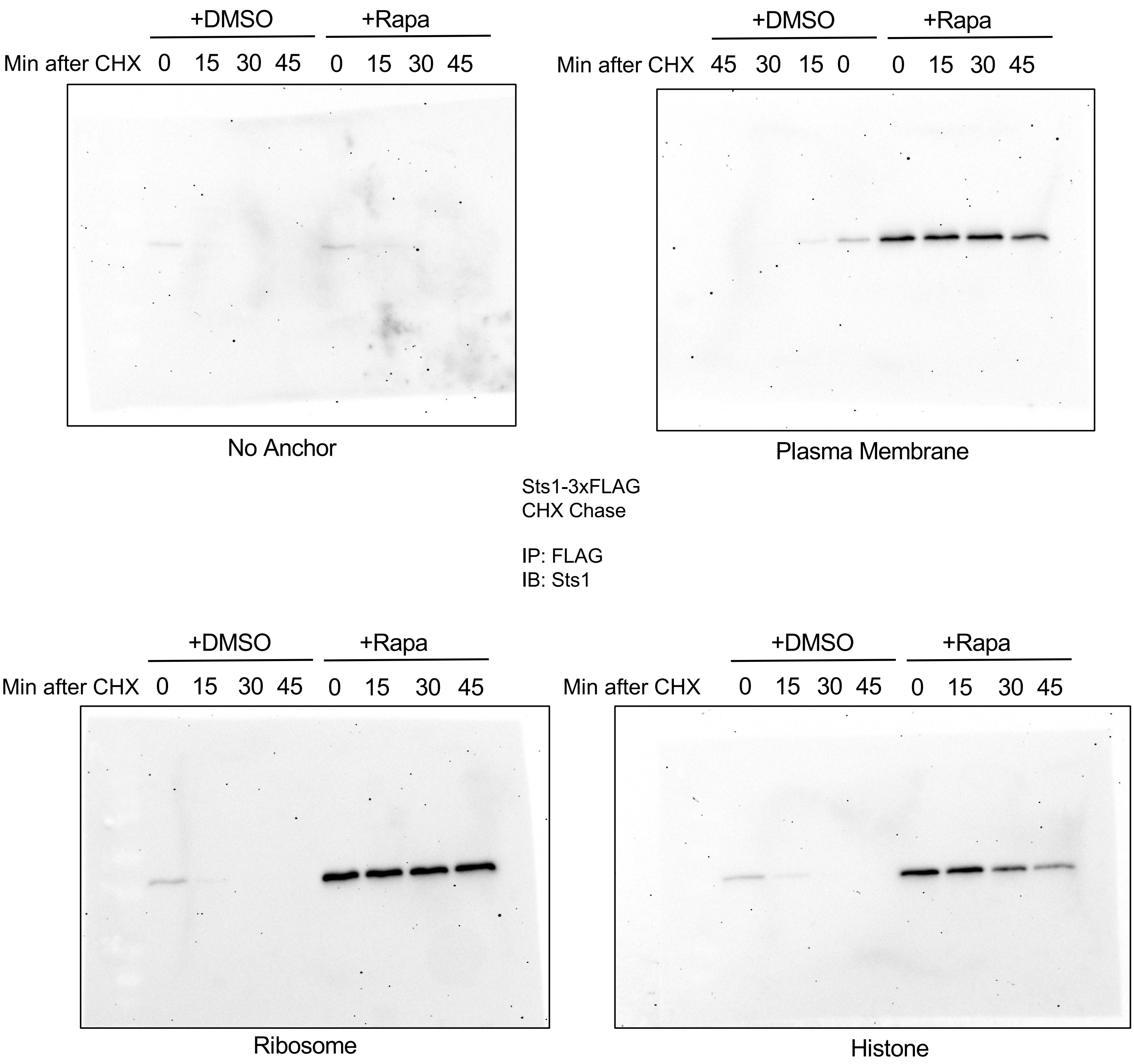

Supplemental Figure 5 (cont.)

H

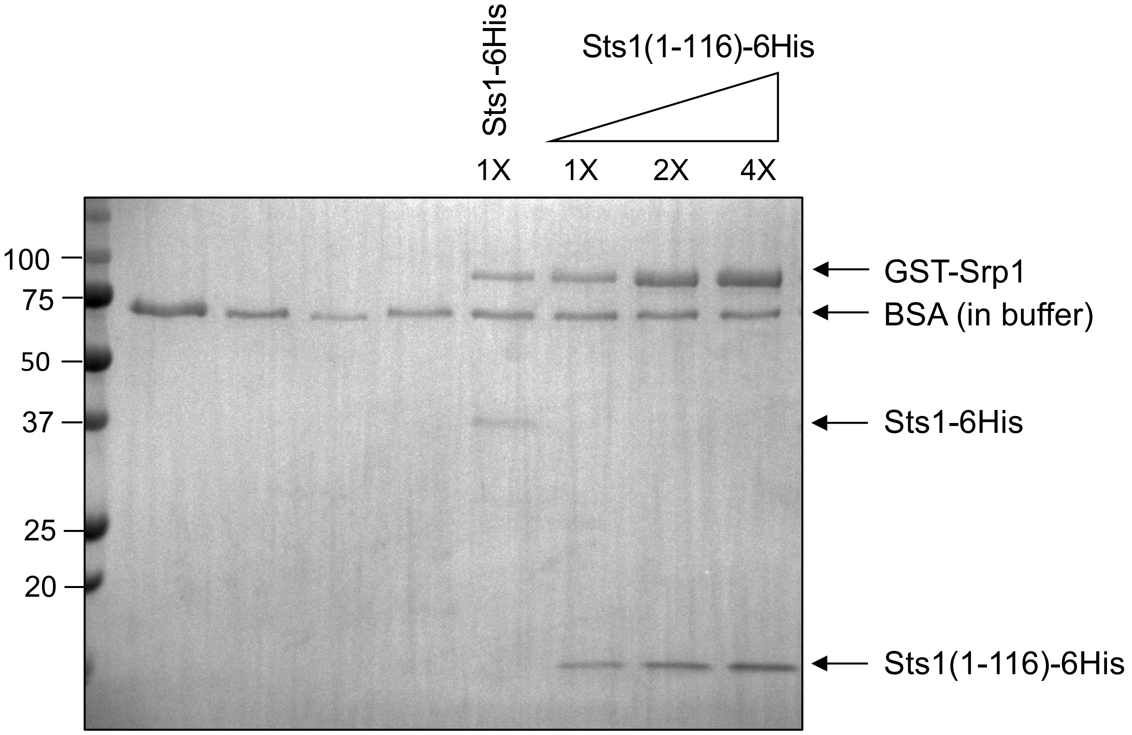

Ponceau S-stained membrane

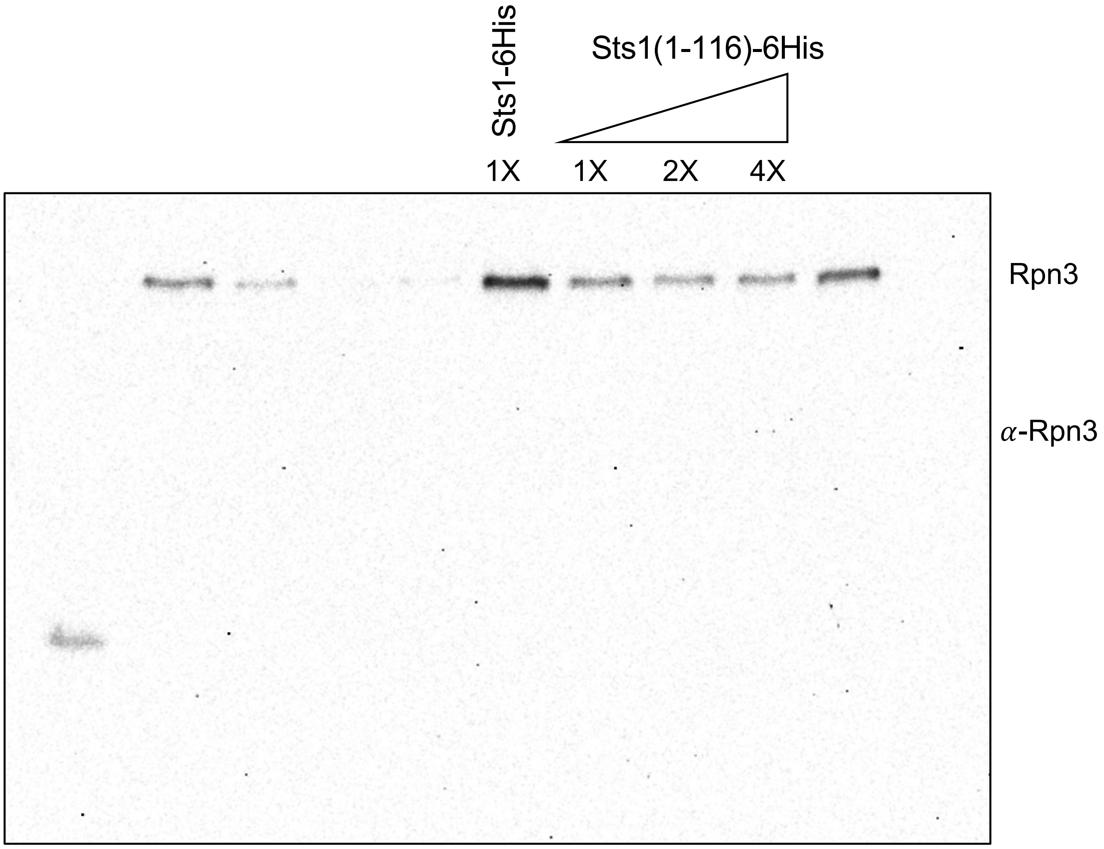

Supplemental Figure 5 (cont.)

I

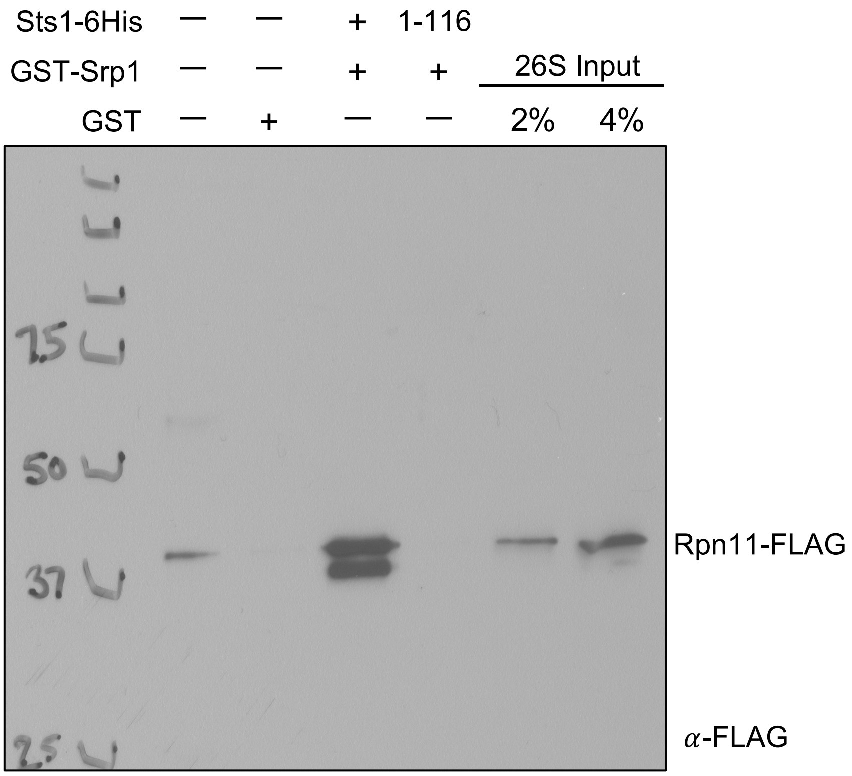

Supplemental Figure 5 (cont.)

J

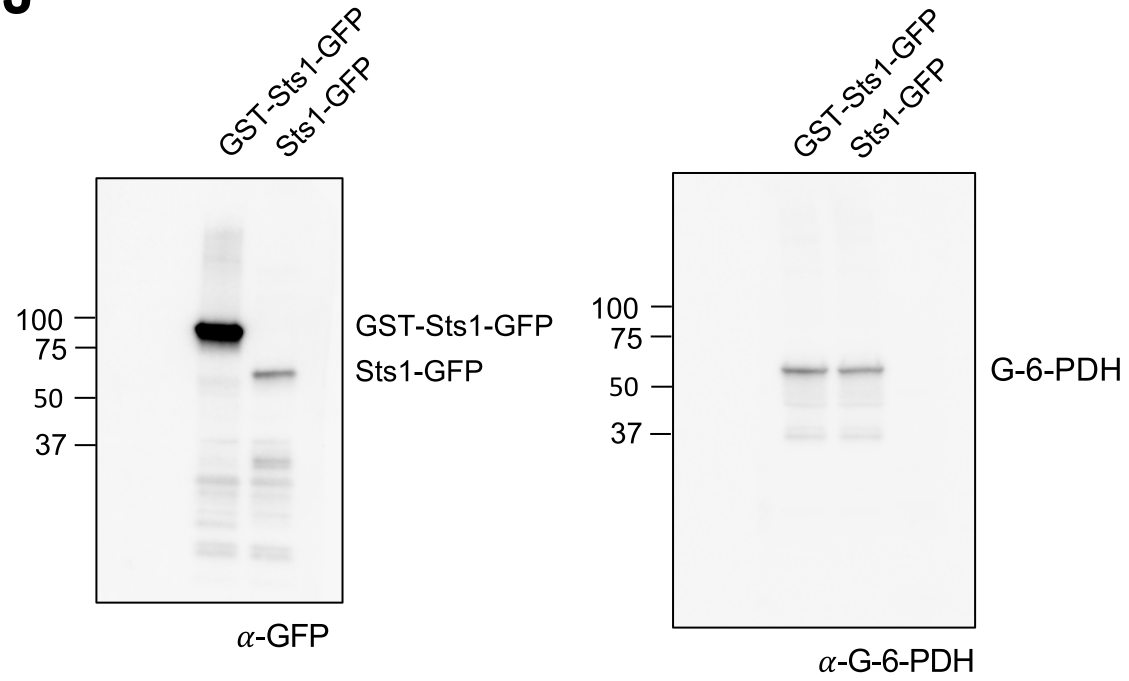

K

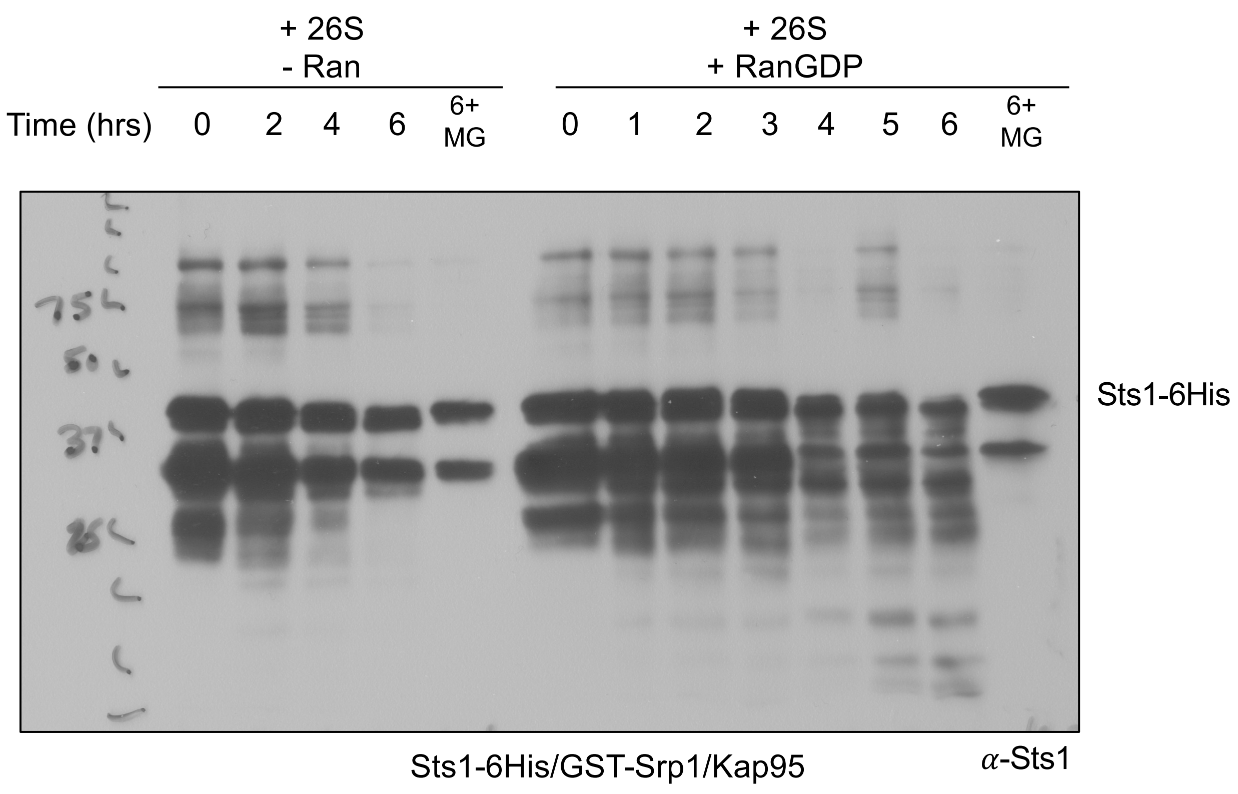

Supplemental Figure 5 (cont.)

L

+DMSO | +Rapa

Time (min) 0 15 30 45 0 15 30 45

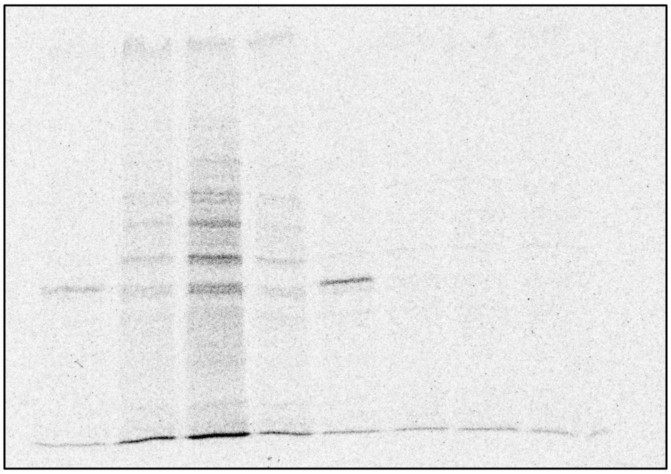

No Anchor

+DMSO | +Rapa

Time (min) 0 15 30 45 0 15 30 45

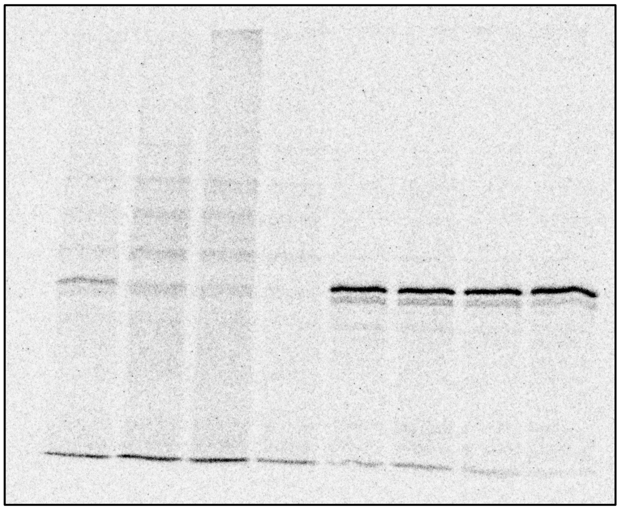

Plasma Membrane

IP: Sts1
Pulse-chase

+DMSO | +Rapa

Time (min) 0 15 30 45 0 15 30 45

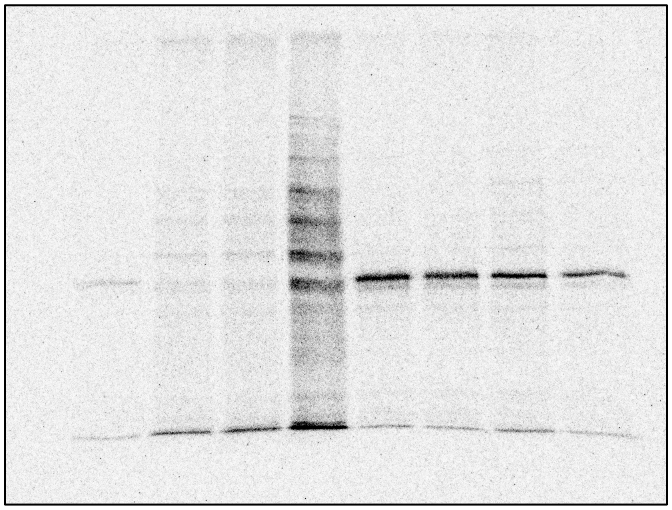

Ribosome

+DMSO | +Rapa

Time (min) 0 15 30 45 0 15 30 45

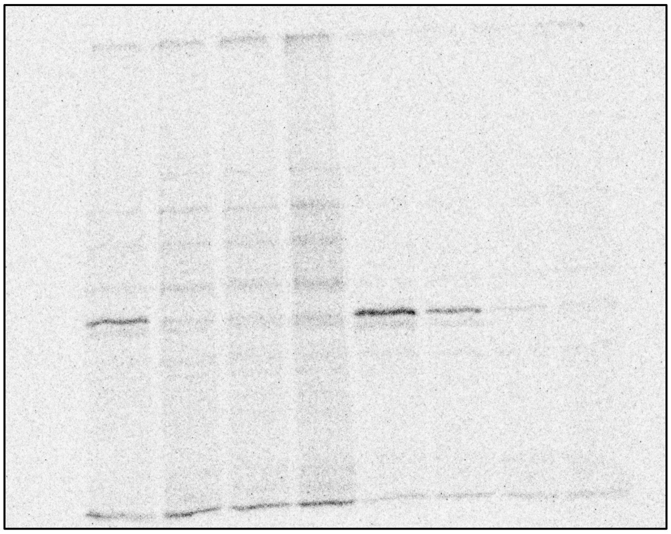

Histone

Supplemental Figure 5 (cont.)

M

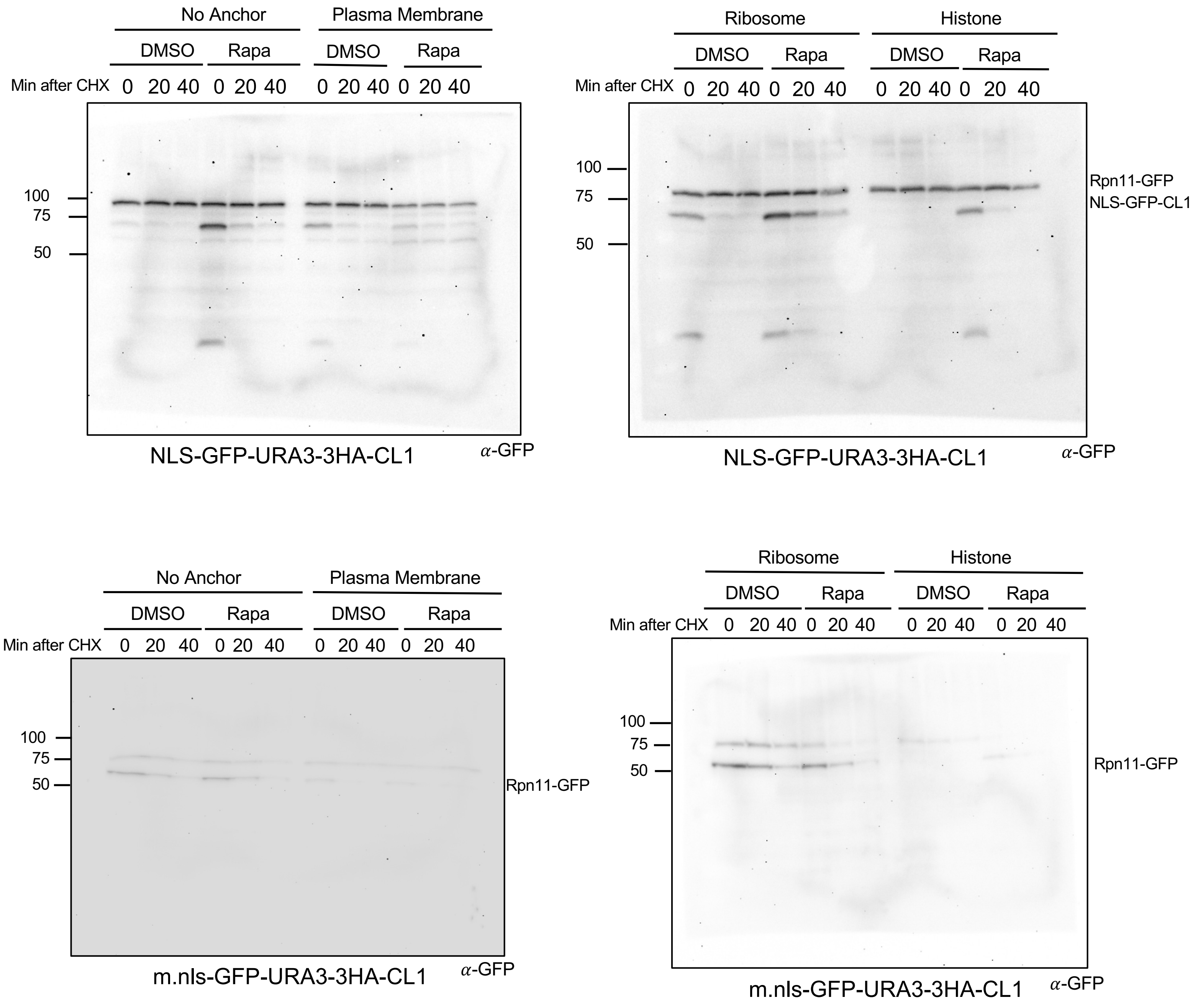

Supplemental Figure 5 (cont.)

N

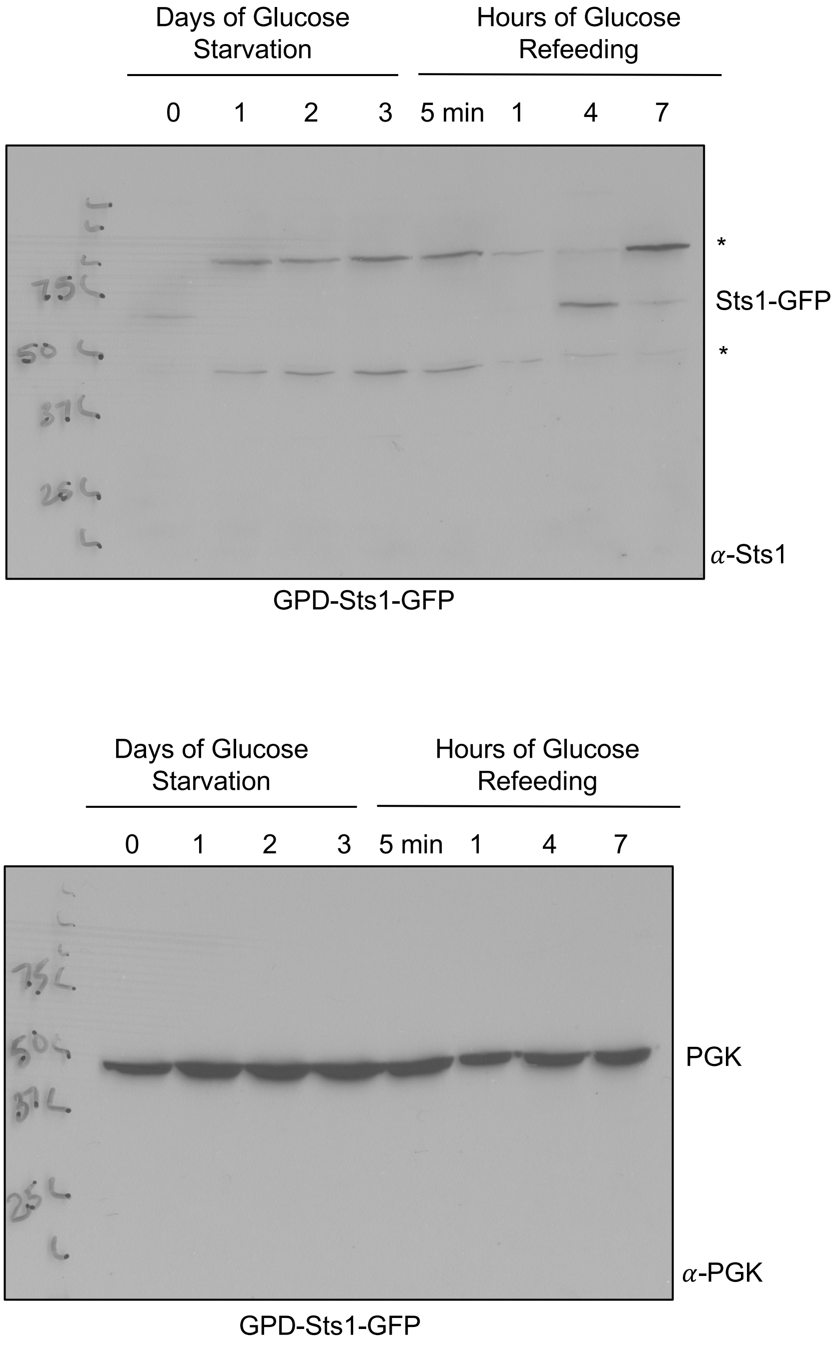

Figure S5. Unedited images presented in this study.

(A) The full-length Western blot of the pull-down assay presented in Figure 1B. Imaged on film. (B) The full-length Western blot of the *in vitro* degradation assay presented in Figure 1C. Imaged on film. (C) The full-length Western blot of the *in vitro* degradation assay presented in Figure 1D. Imaged digitally using a G-Box. (D) The full-length SDS-Page gel, Coomassie Blue-stained, of the pull-down assay presented in Figure 2A. (E) The full-length Western blot of the *in vitro* degradation assay presented in Figure 2B. Imaged on film. (F) The full-length Western blot of the *in vitro* degradation assay presented in Figure 2C. Imaged on film. (G) The full-length Western blots of the *in vivo* Anchor Away degradation assays presented in Figure 3B. From top to bottom, the images represent degradation in the absence of an anchor (No Anchor, left), and the anchors Pma1-FKBP12 (right), Rpl13A-FKBP12 (left), or Htb2-FKBP12 (right). Imaged digitally using a G-Box. (H) The full-length images of the Ponceau-stained membrane presented in Supplemental Figure 1A (top) and the full-length Western blot of the pull-down assay presented in Supplemental Figure 1A (bottom). (I) The full-length Western blot of the pull-down assay presented in Supplemental Figure 1B. Imaged on film. (J) The full-length Western blots of the levels presented in Supplemental Figure 1D. Imaged digitally using a G-Box. (K) The full-length Western blot of the pull-down assay presented in Supplemental Figure 2. Imaged on film. (L) The full-length phosphorimages of the *in vivo* Anchor Away radioactive pulse-chase assays presented in Supplemental Figure 3B. From top to bottom, the images represent degradation in the absence of an anchor (No Anchor, left), the anchor Pma1-FKBP12 (right), the anchor Rpl13A-FKBP12 (left), or Htb2-FKBP12 (right). Imaged using a phosphorimager. (M) The full-length Western blots of the *in vivo* Anchor Away degradation assays presented in Supplemental Figure 3D. The top row represents degradation of NLS-GFP-URA3-3HA-CL1 in the absence of an anchor (No Anchor) or the presence of the anchor Pma1-FKBP12 (left), or in the presence of the anchors Rpl13A-FKBP12 or Htb2-FKBP12 (right). The bottom row represents degradation of m.nls-GFP-URA3-3HA-CL1 in the absence of an anchor (No Anchor) or the presence of the anchor Pma1-FKBP12 (left), or in the presence of the anchors Rpl13A-FKBP12 or Htb2-FKBP12 (right). Imaged digitally using a G-Box. (N) The full-length Western blots of the levels presented in Supplemental Figure 4A. Imaged using film.
