# Supplementary material for: Yeast 26S proteasome nuclear import is coupled to nucleus-specific degradation of the karyopherin adaptor protein Sts1
Source: Sci Rep. 2024 Jan 24;14:2048. doi: 10.1038/s41598-024-52352-5 (PMC10808114; doi:10.1038/s41598-024-52352-5)
Supplement: Supplementary file 6 — Supplementary Tables. [file 41598_2024_52352_MOESM6_ESM.docx]

**Table S1. Yeast Strains Used in This Study**

| Strain | Genotype |  | Source |
| --- | --- | --- | --- |
| MHY500 | *MATa his3-*$\Delta$*200 leu2-3,112 ura3-52 lys2-801 trp1-1 gal2* | | [70] |
| MHY690 | *MATa ade2-1 ura3-1 his3-11 trp1-1 leu2-3,112 can1-100* (W303) | | R. Rothstein |
| MHY5841 | *MATa his3-*$\Delta$*200 leu2-3,112 ura3-52 lys2-801 trp1-1 gal2 RPN11-6xGly-3xFLAG::kanMX6* | | [66] |
| MHY6940 | *MATa his3-*$\Delta$*200 leu2-3,112 ura3-52 lys2-801 trp1-1 gal2 RPN2-mCherry::NAT* | | R. Tomko, MH lab strain |
| MHY8344 | *MATalpha ade2-1 ura3-1 his3-11,15 trp1-1 leu2-3,112 can1-100 TOR1-1 fpr1::natMX RPN11-FRB-GFP::kanMX6* (W303 ) | | [10] |
| MHY8345 | *MATa ade2-1 ura3-1 his3-11,15 trp1-1 leu2-3,112 can1-100 TOR1-1 fpr1::natMX RPN11-FRB-GFP::kanMX6 PMA1-2xFKBP12::TRP1* (W303) | | [10] |
| MHY8346 | *MATa ade2-1 ura3-1 his3-11,15 trp1-1 leu2-3,112 can1-100 TOR1-1 fpr1::natMX RPN11-FRB-GFP::kanMX6 RPL13A-2xFKBP12::TRP1* (W303) | | [10] |
| MHY8347 | *MATa ade2-1 ura3-1 his3-11,15 trp1-1 leu2-3,112 can1-100 TOR1-1 fpr1::natMX RPN11-FRB-GFP::kanMX6 HTB2-FKBP12::HIS3* (W303) | | [10] |
| MHY9579 | *MATalpha sts1Δ::hphMX/pRS316-STS1 (90-9-6) his3-*$\Delta$*200 leu2-3,112 ura3-52 lys2-801 trp1-1 gal2* | | [11] |
| MHY9580 | *MATa sts1Δ::hphMX/pRS316-STS1 (90-9-6) his3-*$\Delta$*200 leu2-3,112 ura3-52 lys2-801 trp1-1 gal2* | | [11] |
| MHY10019 | *MATalpha his3-*$\Delta$*200 leu2-3,112 ura3-52 lys2-801 trp1-1 gal2 atg8*$\Delta$*::hphMX4* | | [64] |
| MHY10148 | *MATalpha his3-*$\Delta$*200 leu2-3,112 ura3-52 lys2-801 trp1-1 gal2 cue5*$\Delta$*::hphMX4* | | J. Li, MH lab strain |
| MHY4464 | *MATa ura3-52 leu2*$\Delta$*1 his3-*$\Delta$*200 trp1*$\Delta$*63 lys2-801 ade2-101 cim3-1* (YPH500) | | M. Funakoshi, MH lab strain |
| MHY11357 | *MATa his3-*$\Delta$*200 leu2-3,112 ura3-52 lys2-801 trp1-1 RPN2-mCherry::NAT sts1*$\Delta$*::hphMX4/*pRS316*-STS1* | | This study |
| MHY12557 | *MATa ade2-1 ura3-1 his3-11,15 trp1-1 leu2-3,112 can1-100 TOR1-1 fpr1::natMX RPN11-FRB-GFP::kanMX6 STS1-3xFLAG::hphMX4* (W303) | | This study |
| MHY12558 | *MATa ade2-1 ura3-1 his3-11,15 trp1-1 leu2-3,112 can1-100 TOR1-1 fpr1::natMX RPN11-FRB-GFP::kanMX6 PMA1-2xFKBP12::TRP1 STS1-3xFLAG::hphMX4* (W303) | | This study |
| MHY12559 | *MATa ade2-1 ura3-1 his3-11,15 trp1-1 leu2-3,112 can1-100 TOR1-1 fpr1::natMX RPN11-FRB-GFP::kanMX6 RPL13A-2xFKBP12::TRP1 STS1-3xFLAG::hphMX4* (W303) | | This study |
| MHY12660 | *MATa ade2-1 ura3-1 his3-11,15 trp1-1 leu2-3,112 can1-100 TOR1-1 fpr1::natMX RPN11-FRB-GFP::kanMX6 HTB2-FKBP12::HIS3 STS1-3xFLAG::hphMX4* (W303) | | This study |
| MHY12579  GFP(+)24-A5 | *MATa his3-*$\Delta$*200 leu2-3,112 ura3-52 lys2-801 trp1-1 gal2 DCP2-mCherry::natMX*  *MATa* *his3*Δ*1* *leu2*Δ*0* *met15*Δ*0* *ura3*Δ*0 STS1-GFP::HIS3MX* | | C. Cheng, MH lab strain  Thermo-Fisher Scientific |

**Table S2: Plasmids Used in this Study**

| Plasmid # | Description | Source |
| --- | --- | --- |
| 19-6-1 | pRS415MET25 | [71] |
| 23-4-7 | pET42b(+) | Novagen |
| 65-6-5 | pET42b(+)-6His-MBP-STS1 | (R. Tomko, MH lab) |
| 78-6-2 | pGEX6P1 | [72] |
| 80-3-7 | pGEX6P1-SRP1 | (J. Ronau MH lab) |
| 90-9-6 | pRS316-STS1 | [11] |
| 91-2-5 | pRS415MET25-STS1-GFP | [11] |
| 91-2-6 | pRS415GPD-STS1-GFP | [11] |
| 91-3-9 | pRS415GPD-sts1(R38D)-GFP | [11] |
| 91-6-4 | pET42b(+)-STS1-6His | [11] |
| 91-9-8 | pRS415GPD-sts1(R65D)-GFP | [11] |
| 94-8-3 | p415MET25-NLS-GFP-URA3-HA-CL1 | [51] |
| 94-9-5 | pGEX6P1-sts1(116-276) | This study |
| 94-9-6 | pGEX6P1-sts1(116-319) | This study |
| 97-5-4 | p415MET25-mutant.NLS-GFP-URA3-HA-CL1 | [51] |
| 100-3-2 | pET42b(+)-SRP1-6His | This study |
| 102-4-9 | pMW172-KAP95 | Anita Corbett |
| 102-5-1 | pGEX4T-1-GST-KAP95 | Anita Corbett |
| 102-5-3 | pET15b-His6-GSP1 | Anita Corbett |
| 109-6-7 | pRS415MET25-STS1-mCherry-FLAG | This study |
| 109-6-8 | pGEX6P1-STS1 | This study |
| 109-7-7 | pRS415MET25-GST-STS1-GFP | This study |
